# Crystal structure of tetra­aqua­(di­methyl­formamide)­tetra­kis­(μ-*N*,2-dioxido­benzene-1-carboximidato)tetra­kis­(μ-tri­methyl­acetato)­tetra­manganese(III)sodiumyttrium–di­methyl­formamide–water (1/8.04/0.62)

**DOI:** 10.1107/S2056989015018216

**Published:** 2015-10-10

**Authors:** Jordan R. Travis, Matthias Zeller, Curtis M. Zaleski

**Affiliations:** a Department of Chemistry, Shippensburg University, 1871 Old Main Dr., Shippensburg, PA 17257, USA; b Department of Chemistry, Youngstown State University, 1 University Plaza, Youngstown, OH 44555, USA

**Keywords:** heterotrimetallic, metallacrown, self-assembled coordination complex, crystal structure

## Abstract

The synthesis and crystal structure for the title compound, [YNaMn_4_(C_7_H_4_NO_3_)_4_(C_5_H_9_O_2_)_4_(H_2_O)_3.76_(C_3_H_7_NO)_0.24_]·8.04C_3_H_7_NO·0.62H_2_O or [Y^III^Na(OTMA)_4_[12-MC_Mn(III)N(shi)_-4](H_2_O)_3.76_(DMF)_0.24_·8.04DMF·0.62H_2_O, where OTMA is tri­methyl­acetate, MC is metallacrown, shi^3−^ is salicyl­hydroximate, and DMF is *N*,*N*-di­methyl­formamide, is reported. The macrocyclic metallacrown consists of an –[Mn^III^–N–O]_4_– ring repeat unit, and the metallacrown captures one Y^III^ ion and one Na^I^ ion in the central cavity on opposite faces of the metallacrown. Overall the metallacrown is domed towards the side of the Na^I^ ion. Both the Y^III^ and Na^I^ ions are eight-coordinate, and the tri­methyl­acetate anions bridge the central Y^III^ to each ring Mn^III^ ion. The ring Mn^III^ ions are six-coordinate with a tetra­gonally distorted octa­hedral geometry.

## Abstract 2

The title compound is an example of a 12-metallacrown-4 self-assembled supra­molecular coordination complex with ring Mn^III^ ions. A Y^III^ ion and Na^+^ ion are captured on opposite sides of the metallacrown cavity, and the Y^III^ ion is tethered to the metallacrown with four tri­methyl­acetate anions.

## Chemical context   

Since 1989 metallacrowns (MCs) have served as an excellent example of the controllable self-assembly of supra­molecular coordination complexes (Mezei *et al.*, 2007 ▸). Considered the structural and functional inorganic analogues to crown ethers, metallacrowns self-assemble in solution to form coordination complexes with multiple metal centers. Not only can homometallic complexes be synthesized, but heterobimetallic and heterotrimetallic metallacrowns can also be prepared through one-step reactions (Mezei *et al.*, 2007 ▸; Azar *et al.*, 2014 ▸). The deliberate formation of supra­molecular coordination complexes, especially those with multiple metal types, remains a synthetic challenge (Cook & Stang, 2015 ▸; Saalfrank *et al.*, 2008 ▸); however, metallacrowns provide a class of mol­ecules that allows the investigation of the formation of multi-metal supra­molecular coordination complexes.

Recently we reported the first synthetic strategy for heterotrimetallic metallacrowns: *Ln* ^III^ *M*(OAc)_4_[12-MC_Mn(III)N(shi)_-4], where *Ln* ^III^ is Pr^III^ to Yb^III^ (except Pm^III^) and Y^III^, *M* is Na^I^ or K^I^, ^−^OAc is acetate, and shi^3−^ is salicyl­hydroximate (Azar *et al.*, 2014 ▸). In the previous report, we demonstrated the ability to systematically replace the central metal ions; however, the metallacrown framework has other points of alteration, in particular the bridging carboxyl­ate anion. In these alkali metal–lanthanide–manganese ion complexes, four acetate anions serve as bridges between the central lanthanide ion and the ring Mn^III^ ions. Potentially the acetate anions could be replaced with other carboxyl­ate monoanions.

Herein we report the synthesis and crystal structure of Y^III^Na(OTMA)_4_[12-MC_Mn(III)N(shi)_-4](H_2_O)_3.76_(DMF)_0.24_·8.04DMF·0.62H_2_O, (**1**), where OTMA is tri­methyl­acetate and DMF is *N*,*N*-di­methyl­formamide. This metallacrown demonstrates the ability to vary the bridging carboxyl­ate monoanion of this heterotrimetallic class of metallacrowns.
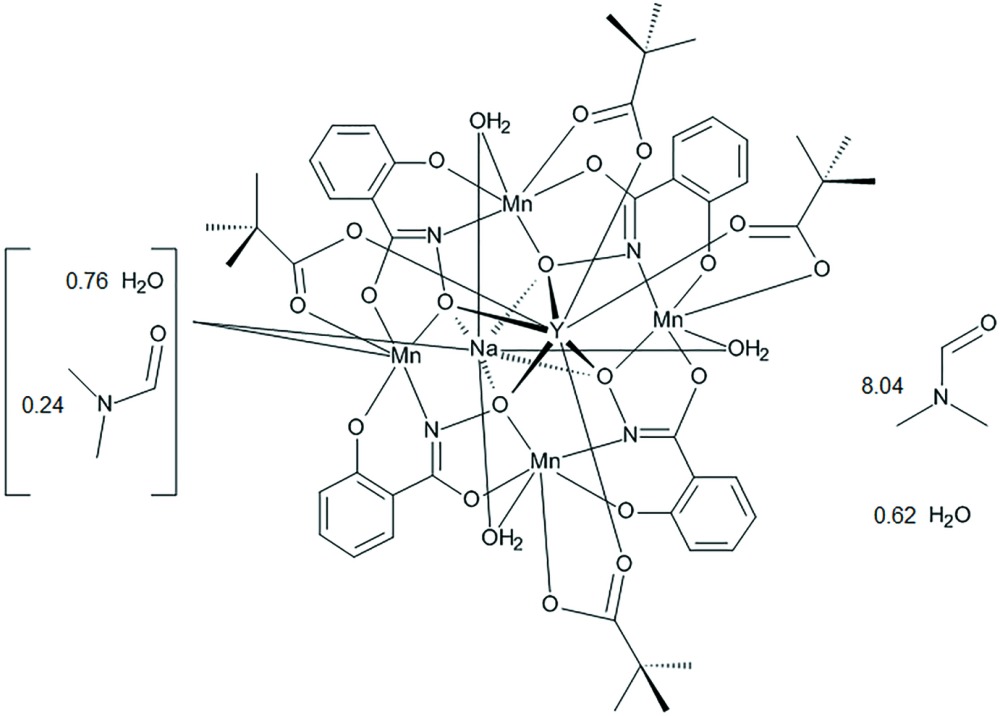


## Structural commentary   

**Figure 1:**
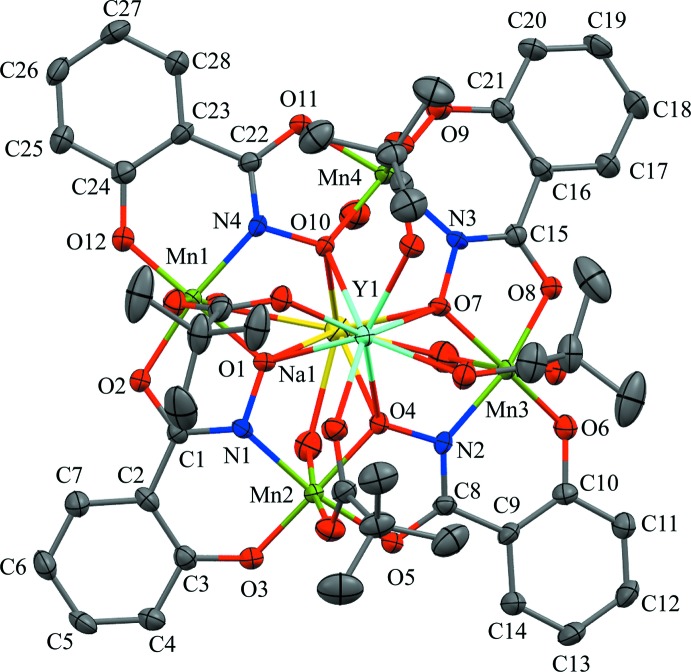
The mol­ecular structure of (**1**) in top view with displacement ellipsoids at the 50% probability level. For clarity, H atom and lattice solvent mol­ecules have been omitted, and only atom labels for all non-H atoms of the 12-MC-4 framework have been provided. Color scheme: aqua – Y^III^, green – Mn^III^, yellow – Na^+^, red – oxygen, blue – nitro­gen, and gray – carbon.

The structure of the title compound Y^III^Na(OTMA)_4_[12-MC_Mn(III)N(shi)_-4](H_2_O)_3.76_(DMF)_0.24_·8.04DMF·0.62H_2_O, (**1**), is based on the typical [12-MC_Mn(III)N(shi)_-4] core. Four shi^3−^ framework ligands and four Mn^III^ ions self-assemble to form an overall square geometry with a –[Mn-N-O]_4_– repeat unit. The MC ring forms a central cavity with a pseudo-fourfold rotation axis that is capable of binding central metal ions, in this structure an Y^III^ ion and a Na^I^ ion. The two ions are bound on opposite faces of the MC, and the metallacrown is slightly domed with the Y^III^ ion residing on the convex side of the central cavity and the Na^I^ ion residing on the underside of the dome. The Y^III^ ion is also connected to the MC core by four tri­methyl­acetate monoanions that serve to bridge the Y^III^ ion to each ring Mn^III^ ion. The mol­ecular structure is shown in Figs. 1 ▸ and 2 ▸.

The ring Mn^III^ ions and the central Y^III^ ion are assigned a 3+ oxidation state based on average bond lengths, calculated bond-valence-sum (BVS) values (Liu & Thorp, 1993 ▸), and overall mol­ecular charge considerations. For Mn1, Mn2, Mn3, and Mn4, the average bond lengths are 2.05, 2.04, 2.06, and 2.05 Å, respectively, and the calculated BVS values for Mn1–Mn4 are 3.04, 3.06, 3.07, and 3.05 v. u., respectively. In addition, each Mn^III^ possesses elongated axial bond lengths, which would be expected for a high-spin *d* ^4^ ion. The Y1 ion has an average bond length and BVS value of 2.35 Å and 3.32 v. u., respectively. Mol­ecular charge neutrality considerations also support the assigned oxidation states as the four shi^3−^ ligands and four tri­methyl­acetate monoanions (total 16- charge) are balanced by the presence of four Mn^III^ ions, one Y^III^ ion, and one Na^I^ ion (total 16+ charge).

The Y^III^ ion is eight-coordinate with a distorted square anti­prismatic geometry. The first coordination sphere is provided by two planes of four oxygen atoms each. One plane consists of four carboxyl­ate oxygen atoms from the bridging tri­methyl­acetate anions, and the second plane is formed by four oxime oxygen atoms of the MC ring. The Y^III^ ion lies closer to the mean plane of the carboxyl­ate oxygen atoms (O_car_MP), 1.07 Å, than the mean plane of the oxime oxygen atoms (O_ox_MP), 1.57 Å. Also, the two planes are twisted relative to each other with an average skew angle of 50.02^o^ about the Y^III^ ion (AlDamen *et al.*, 2008 ▸, 2009 ▸). The skew angles were calculated with the program *Mercury* (Macrae *et al.*, 2006 ▸) and determined as previously described (Azar *et al.*, 2014 ▸). For an ideal square-prismatic geometry, the skew angle is 0^o^, while for an ideal square-anti­prismatic geometry, the skew angle is 45^o^. Given the measured skew angle and the placement of the Y^III^ ion relative to the two planes of oxygen atoms, the best description of the geometry is distorted square anti­prismatic.

The Na^I^ ion is eight-coordinated with a severely distorted square-anti­prismatic geometry. As in the Y^III^ ion, the first coordination sphere is supplied by two planes of four oxygen atoms each. One plane is composed of the four oxime oxygen atoms of the MC ring, and the second plane consists of oxygen atoms from solvent mol­ecules. Three of the four coordination sites are occupied by water mol­ecules, while a water mol­ecule and DMF mol­ecule are disordered over the fourth site with an occupancy ratio of 0.758 (8):0.242 (8) (complete refinement details are given below). The Na^I^ ion lies closer to the mean plane of the solvent oxygen atoms (O_solvent_MP), 0.67 Å, than the mean plane of the oxime oxygen atoms, 1.97 Å. Also, the two planes are twisted relative to each other with an average skew angle of 29.18^o^ about the Na^I^ ion. Lastly, the solvent oxygen atoms bridge the central Na^I^ ion to the ring Mn^III^ ions. The water and DMF mol­ecules disordered over the coordin­ation site to the Na^I^ ion bridge the Na^I^ ion to Mn4.

Each ring Mn^III^ is six-coordinate with a tetra­gonally distorted octa­hedral geometry. The equatorial plane is comprised of a six-membered chelate ring and a *trans* five-membered chelate ring. The six-membered chelate ring is formed from the oxime nitro­gen atom and the phenolate oxygen atom of one shi^3−^ ligand, and the five-membered chelate ring is formed from the oxime oxygen atom and the carbonyl oxygen atom of a second shi^3−^ ligand. Each Mn^III^ ion possesses an elongated axial axis, which is composed of a carboxyl­ate oxygen atom from a bridging tri­methyl­acetate anion and a bridging solvent oxygen atom from either a water or a DMF mol­ecule. The Mn^III^—O_solvent_ bond lengths are rather long (2.4–2.5 Å), which is likely due to the simultaneous coordination to the central Na^I^ ion.

The metallacrown is slightly domed toward the central Na^I^ ion. As previously reported, the doming effect is not likely due to the presence of either central metal ion, but likely due to the displacement of each ring Mn^III^ ion from the equatorial mean plane of its first coordination sphere ligand atoms (Azar *et al.*, 2014 ▸). For (**1**), the average distance of the ring Mn^III^ ions above the equatorial ligand atom mean plane is 0.15 Å. Another indication of the doming effect in the MC is the angle between the axial carboxyl­ate oxygen atom, the ring Mn^III^ ion, and the calculated centroid of the oxime oxygen atoms (*Mercury;* Macrae *et al.*, 2006 ▸). In a planar MC, this angle would be 90^o^. For the title compound, the average angle about the Mn^III^ ions is 101.74^o^, which indicates that the MC is slightly domed.

In addition to the MC, several solvent mol­ecules are located in the lattice some of which are only partially occupied (complete refinement details are given below). Three different DMF mol­ecules are flipped disordered over two sites, one DMF mol­ecule is disordered over two sites with different orientations, and two DMF mol­ecules are partially occupied. In addition, the disordered water/DMF binding site of the Na^I^ ion is correlated to two DMF mol­ecules, one of which is disordered over two sites with different orientations, and to two partially occupied water mol­ecules. Overall there is a total of 8.04 DMF and 0.62 water mol­ecules located in the lattice.

## Supra­molecular features   

**Figure 3:**
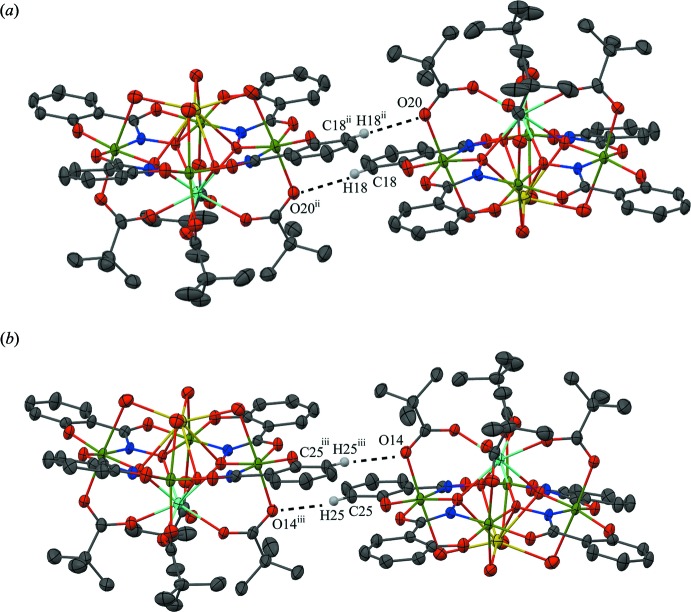
Inter­molecular C—H⋯O inter­actions between adjacent metallacrowns. For clarity the inter­actions have been divided into two sections (*a*) and (*b*), only the H atoms (white) involved in the inter­actions have been included, and only the atoms involved in the inter­actions have been labelled. See Fig. 1 ▸ for display details. [Symmetry codes: (ii) −*x* + 2, −*y* + 1; (iii) −*x* + 2, −*y* + 1, −*z*.]

**Figure 4:**
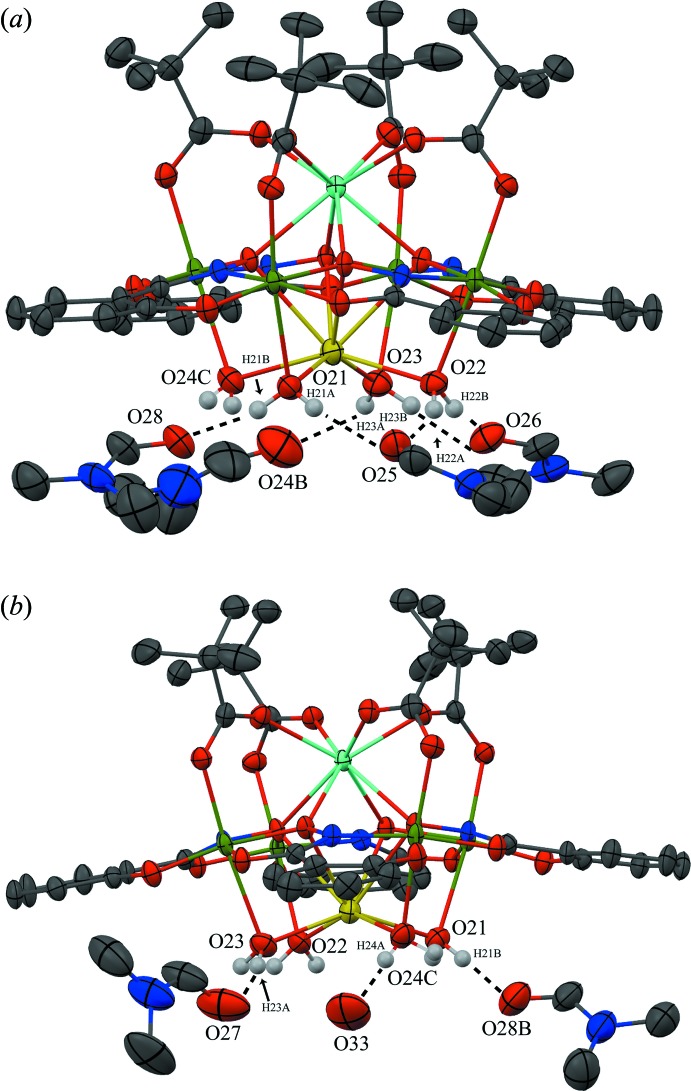
Inter­molecular hydrogen bonding between the water mol­ecules coordin­ating to the Na^+^ ion and the water and DMF mol­ecules of the lattice. For clarity the hydrogen bonding has been divided into two sections (*a*) and (*b*), only the H atoms (white) involved in the hydrogen bonding have been included, and only the atoms involved in the hydrogen bonding have been labelled. See Fig. 1 ▸ for display details.

**Figure 5:**
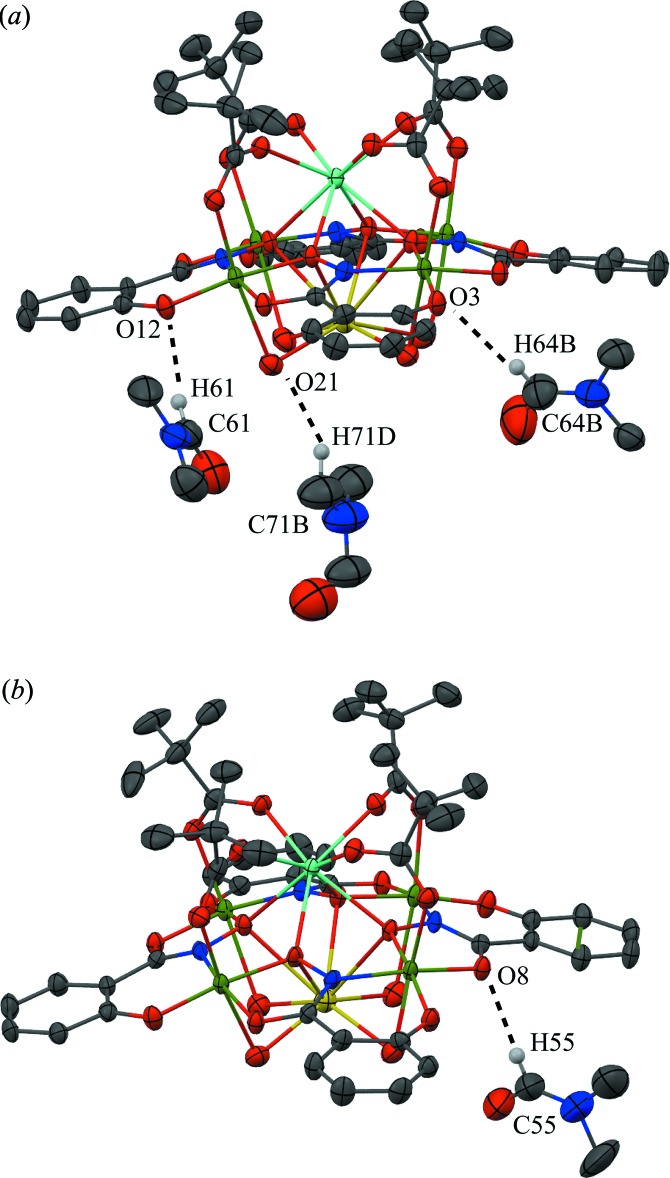
Inter­molecular C—H⋯O inter­actions between the metallacrown and the DMF mol­ecules of the lattice. For clarity the inter­actions have been divided into two sections (*a*) and (*b*), only the H atoms (white) involved in the inter­actions have been included, and only the atoms involved in the inter­actions have been labelled. See Fig. 1 ▸ for display details.

**Figure 6:**
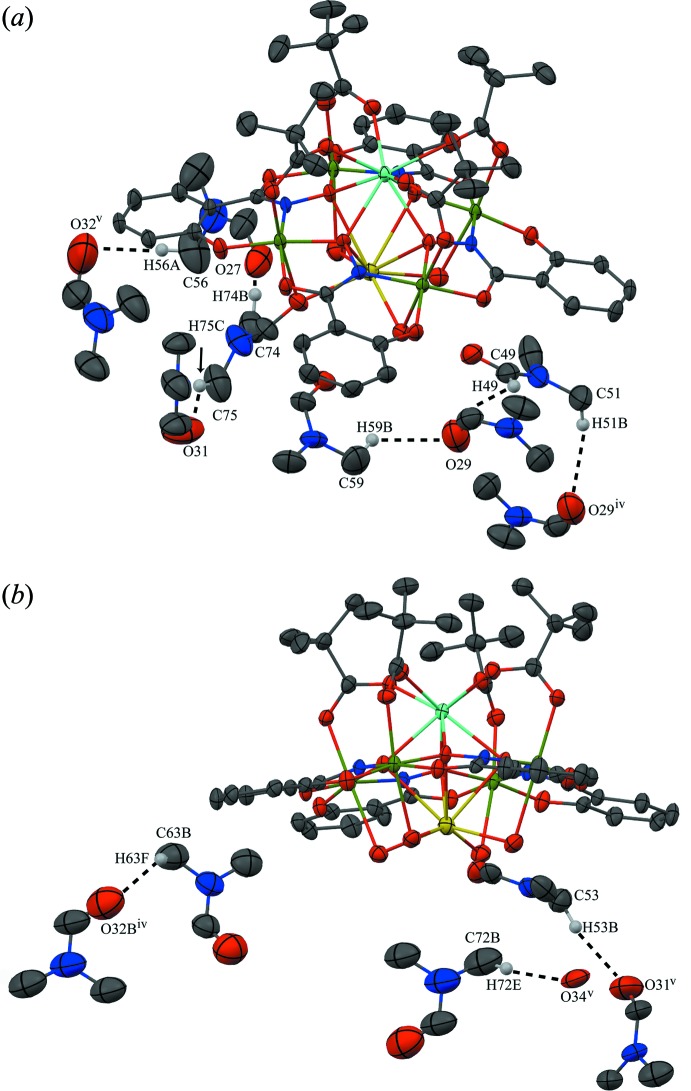
Inter­molecular C—H⋯O inter­actions between adjacent water and DMF mol­ecules. For clarity the inter­actions have been divided into two sections (*a*) and (*b*), only the H atoms (white) involved in the inter­actions have been included, and only the atoms involved in the inter­actions have been labelled. See Fig. 1 ▸ for display details. [Symmetry codes: (iv) −*x* + 1, −*y* + 1, −*z*; (v) −*x* + 1, −*y* + 1, −*z* + 1.]

No strong directional inter­molecular inter­actions are observed between the Y^III^Na(OTMA)_4_[12-MC_Mn(III)N(shi)_-4](H_2_O)_3.76_(DMF)_0.24_ mol­ecules, but inter­molecular C—H⋯O inter­actions exist between adjacent metallacrowns (Table 1 ▸). The inter­actions exist between the carboxyl­ate oxygen atoms (O14 and O20) of the tri­methyl­acetate anions and the benzene carbon atoms (C18 and C25) of the shi^3−^ ligands on adjacent metallacrowns (Fig. 3 ▸). In addition, the water mol­ecules (O21, O22, O23, and O24*C*) coordinating to the Na^I^ ion are hydrogen bonded to several lattice water and DMF mol­ecules (Fig. 4 ▸), and the lattice DMF mol­ecules inter­act with the MC mol­ecule through C—H⋯O inter­actions (Fig. 5 ▸). The C—H⋯O inter­actions occur between either a phenolate oxygen atoms (O3 and O12) of shi^3−^ ligands, a carboxyl­ate oxygen atom (O8) of a shi^3−^ ligand, or a coordinating water oxygen atom (O21) and carbonyl carbon atoms (C55, C61, and C64*B*) or a methyl carbon atom (C71*B*) of lattice DMF mol­ecules (Fig. 5 ▸). Lastly, several C—H⋯O inter­actions exist between adjacent solvent mol­ecules (Fig. 6 ▸). The carbonyl (C49) or methyl (C51, C53, C56, C59, C63*B*, C72*B*, C74, and C75) carbon atoms of DMF mol­ecules inter­act with either an oxygen atom (O34) of a lattice water mol­ecule or carbonyl oxygen atoms (O27, O29, O31, O32, and O32*B*) of lattice DMF mol­ecules. The hydrogen bonding and weak C—H⋯O inter­actions, in addition to pure van der Waals forces, contribute to the overall packing of the mol­ecules.

## Database survey   

The crystal structure of one other yttrium-based heterotrimetallic 12-MC-4 has been reported: Y^III^Na(OAc)_4_[12-MC_Mn(III)N(shi)_-4](H_2_O)_4_·6DMF, **2** (Azar *et al.*, 2014 ▸). In the title compound (**1**), tri­methyl­acetate anions bridge the central Y^III^ ion to the ring Mn^III^ ions, while in the previously reported compound (**2**) acetate anions bridge the Y^III^ ion and the Mn^III^ ions. Also for the previously reported compound (**2**), there are two independent MCs in each unit cell; thus, the labels (**2A**) and (**2B**) will be used to distinguish the two MCs. The replacement of acetate for tri­methyl­acetate does not severely distort the [12-MC_Mn(III)N(shi)_-4] framework. Comparing the two carboxyl­ate monoanion structures, several key features of both MCs are very similar (Table 2 ▸). These features were calculated and measured using the program *Mercury* (Macrae *et al.*, 2006 ▸) and in the same manner as previously described (Azar *et al.*, 2014 ▸). Comparable measured values for the MC cavity radii, average adjacent Mn^III^—Mn^III^ distances, cross cavity Mn^III^—Mn^III^ distances, and cross cavity oxime oxygen (O_ox_—O_ox_) distances demonstrate that the [12-MC_Mn(III)N(shi)_-4] framework is not significantly affected by the identity of the bridging carboxyl­ate anion. In addition, the determined metrics of the central Y^III^ ions and Na^+^ ions are very similar in both (**1**) and (**2**) (Table 2 ▸). The greatest deviations between the structures is the distance of the Na^I^ ion from the mean plane of the solvent oxygen atoms. This is likely due to the difference in the first coordination sphere of the Na^I^ ions. In (**2A**) and (**2B**) only water mol­ecules bind to the Na^I^ ions, while in (**1**) a mixture of water and DMF mol­ecules bind to the Na^I^ ion.

The identity of the bridging ligand does not significantly alter the domed feature of the metallacrown. As stated in the *Structural commentary* for (**1**), the average distance of the ring Mn^III^ ions above the equatorial ligand atom mean plane is 0.15 Å, and the average angle about the Mn^III^ ions with respect to the axial carboxyl­ate oxygen atom and the calculated centroid of the oxime oxygen atoms is 101.74^o^. For (**2A**) and (**2B**), the Mn^III^ ions in both structures are on average 0.17 Å above the equatorial ligand atom mean plane, and the average angles about the Mn^III^ ions with respect to the axial carboxyl­ate oxygen atom and the calculated centroid of the oxime oxygen atoms are 102.31 and 102.04^o^, respectively.

## Synthesis and crystallization   

The title compound (**1**) was synthesized by first mixing yttrium(III) nitrate hexa­hydrate (0.125 mmol), sodium tri­methyl­acetate hydrate (4 mmol based on an assumption of three waters of hydration), and salicyl­hydroxamic acid (2 mmol) in 10 mL of DMF resulting in a cloudy, white mixture. In a separate beaker, manganese(II) acetate tetra­hydrate (2 mmol) was dissolved in 10 mL of DMF resulting in an orange–red solution. The two solutions were mixed resulting in a dark-brown solution and then allowed to stir overnight. The solution was then filtered to remove a dark-brown precipitate, which was discarded. Slow evaporation of the dark-brown filtrate yielded X-ray quality black/dark-brown crystals after 9 days. The yield was 20% based on yttrium(III) nitrate hexa­hydrate.

## Refinement   

Crystal data, data collection and structure refinement details are summarized in Table 3 ▸. The following low angle reflections were affected by the beam stop and were omitted from the refinement: 1 0 0, 0 1 0, 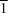 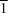 1, and 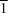 1 0. For all of the disordered solvate water and DMF mol­ecules, neighboring atoms were restrained to have similar *U*_ij_ components of their ADPs if closer than 1.7 Å (SIMU restraints in *SHELXL*).

The geometries of the DMF mol­ecules associated with N7, N8*B*, N9, N9*B*, N10, N10*B*, N11, N12, N12*B*, N13, and N13*B* were restrained to be similar to the DMF mol­ecule associated with N5 (esd = 0.02 Å). For the DMF mol­ecules associated with N7*B* and N11*B*, the geometries were restrained to be similar to the DMF mol­ecule associated with N5 (esd = 0.001 Å). For the DMF mol­ecules associated with N8*B*, N11*B*, and N13*B*, the carbon, oxygen, and nitro­gen atoms were restrained to lie in the same plane (e.s.d. = 0.01 Å^3^).

A water mol­ecule (O24*C*) and DMF mol­ecule associated with N13 are disordered over a binding site to Na1. The atoms O24 and O24*C* were given identical coordinates, and to avoid correlation of the thermal parameters, the ADPs of O24 and O24*C* were constrained to be identical. Subject to these and the above conditions, the occupancy ratio of the disordered water and DMF mol­ecules refined to 0.758 (8) to 0.242 (8). Correlated to the occupation of the binding site to Na1 is a DMF mol­ecule associated with N13*B* and a DMF mol­ecule associated with N7 that is disordered over two sites with different orientations. Subject to the above restraints, the occupancy ratio of the DMF mol­ecule associated with N13*B* refined to 0.252 (5), and the occupancy ratio of the disordered DMF mol­ecule associated with N7 refined to 0.748 (5):0.252 (5). In addition, two partially occupied water mol­ecules associated with O33 and O34 are correlated to these water and DMF mol­ecules. The occupancy of the water mol­ecule of O33 and the water mol­ecule of O34 are 0.257 (14) and 0.361 (13), respectively.

Several DMF mol­ecules are disordered, and the above restraints were used to model the data. The DMF mol­ecule associated with N8 is flipped disordered over two sites, and the occupancy ratio refined to 0.813 (7):0.187 (7). The DMF mol­ecule associated with N9 is flipped disordered over two sites, and the occupancy ratio refined to 0.813 (7):0.187 (7). The DMF mol­ecule associated with N10 is disordered over two sites with different orientations, and the occupancy ratio refined to 0.795 (6):0.205 (6). The DMF mol­ecule associated with N11 is flipped disordered over two sites, and the occupancy ratio refined to 0.790 (9):0.210 (9). Two DMF mol­ecules associated with N12 and N12*B* are partially occupied. The occupancy of the DMF mol­ecule N12 and the DMF mol­ecule 12B are 0.662 (8) and 0.129 (7), respectively.

For the water mol­ecules, the oxygen–hydrogen bond lengths were restrained to 0.84 (2) Å. The hydrogen–hydrogen distances for the water mol­ecules associated with O24, O33, and O34 were restrained to 1.36 (2) Å. For the water mol­ecule O24*C*, the hydrogen atoms were restrained to a distance of at least 2.90 (2) Å from Na1. For the water mol­ecules associated with O33 and O34, the hydrogen atoms were refined as riding on the oxygen atoms.

For the methyl group carbon atoms C56*B*, C62*B*, C63*B*, C69, C69*B*, C71*B*, C72*B*, C74, C74*B*, C75, and C75*B*, hydrogen atoms were placed in tetra­hedral positions with an ideal staggered geometry (AFIX 33). All other methyl group hydrogen atoms were allowed to rotate. All other hydrogen atoms were placed in calculated positions and refined as riding on their carrier atoms with C—H distances of 0.95 Å for *sp* ^2^ carbon atoms and 0.98 Å for methyl carbon atoms. The *U*_iso_ values for hydrogen atoms were set to a multiple of the value of the carrying carbon atom (1.2 times for *sp* ^2^-hybridized carbon atoms or 1.5 times for methyl carbon atoms and water oxygen atoms).

Several larger than desired residual electron density peaks remain after refinement of the data, which is typical for this class of compounds. The origin of these peaks is usually caused either by minor twinning, excessive twinning with multiple components that is beyond what can be completely handled with current integration and absorption correction software, pseudosymmetry (and correlation), or additional disorder not defined well enough to be modeled. In the case of the presented structure, the residual electron density is mostly due to additional disorder. The 3^rd^, 4^th^, 5^th^ and 7^th^ largest residual electron density peaks are due to alternative positions of manganese atoms of a minor moiety of the metallacrown unit (whole mol­ecule disorder). The height of these peaks, 1.3 to 1.2 electrons per Å^3^, indicate the presence of less than 5% of the second moiety, and most other atoms (carbon, nitro­gen, and oxygen) are not resolved. The 2^nd^ largest residual density peak (1.71 electrons per Å^3^) is located close to the yttrium atom and is within the typical range of residual electron density peaks close to heavy atoms. The two remaining residual electron density peaks, the largest (1.73 electrons per Å^3^) and 6^th^ largest (1.23 electrons per Å^3^) are due to minor twinning by a 180.0 degree rotation about the 1 1 0 reciprocal lattice direction (twin law 0.215 0.785 −0.203, 1.215 −0.215 −0.203, 0 0 −1). Refinement as a non-merohedric twin does reduce these peaks to 1.14 and 0.71 electrons per Å^3^, respectively; however, the *R*1 value slightly increases to 0.0553 from 0.0525. Also, the other larger residual electron density peaks (see above) are not improved by inclusion of twinning, nor is the structural model in any way changed. Considering the very minor effect, non-merohedric twinning was not used.

## Supplementary Material

Crystal structure: contains datablock(s) I. DOI: 10.1107/S2056989015018216/bg2568sup1.cif

Structure factors: contains datablock(s) I. DOI: 10.1107/S2056989015018216/bg2568Isup2.hkl

CCDC reference: 1428526

Additional supporting information:  crystallographic information; 3D view; checkCIF report

## Figures and Tables

**Figure 2:**
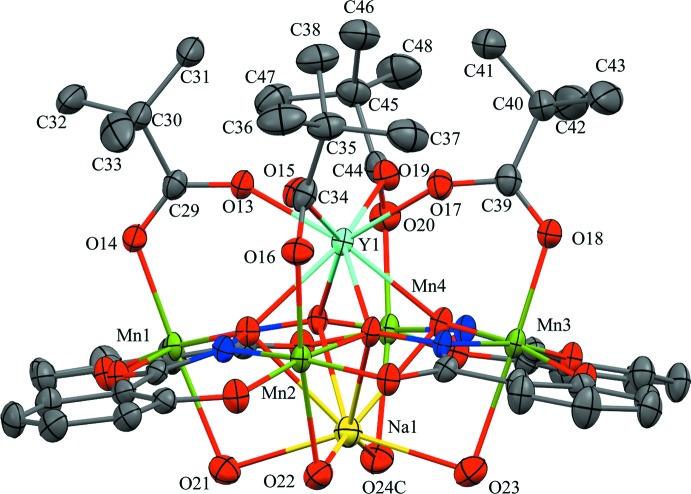
The mol­ecular structure of (**1**) in side view. For clarity, only atom labels for all non-H atoms of the tri­methyl­acetate anions and the coordinating water mol­ecules and of the metal ions have been provided. For the solvent coordination site to Mn4, a water mol­ecule and DMF mol­ecule are disordered with an occupancy ratio of 0.758 (8):0.242 (8). Only the water mol­ecule is displayed. See Fig. 1 ▸ for display details.

**Table 1:** Hydrogen-bond geometry (, )

*D*H*A*	*D*H	H*A*	*D* *A*	*D*H*A*
C18H18O20^i^	0.95	2.60	3.359(5)	137
C25H25O14^ii^	0.95	2.59	3.374(5)	141
C49H49O29	0.95	2.58	3.180(8)	121
C51H51*B*O29^iii^	0.98	2.56	3.376(9)	141
C53H53*B*O31^iv^	0.98	2.48	3.377(9)	152
C55H55O8	0.95	2.36	3.098(8)	135
C56H56*A*O32^iv^	0.98	2.56	3.499(17)	162
C59H59*B*O29	0.98	2.56	3.262(11)	129
C61H61O12	0.95	2.52	3.457(8)	169
C63*B*H63*F*O32*B* ^iii^	0.98	2.53	3.34(6)	140
C64*B*H64*B*O3	0.95	2.50	3.40(3)	157
C71*B*H71*D*O21	0.98	2.60	3.41(5)	141
C72*B*H72*E*O34^iv^	0.98	2.36	3.31(7)	163
C74H74*B*O27	0.98	2.27	2.87(3)	119
C75H75*C*O31	0.98	2.15	2.99(3)	143
O21H21*A*O25	0.82(2)	2.00(3)	2.767(4)	155(5)
O21H21*B*O28	0.83(2)	2.05(3)	2.792(5)	148(5)
O21H21*B*O28*B*	0.83(2)	1.87(3)	2.70(2)	172(5)
O22H22*A*O25	0.84(2)	1.96(3)	2.727(4)	151(5)
O22H22*B*O26	0.83(2)	1.93(3)	2.688(4)	151(5)
O23H23*A*O27	0.84(2)	2.06(3)	2.871(7)	164(5)
O23H23*A*O24*B*	0.84(2)	2.06(5)	2.696(19)	132(5)
O23H23*B*O26	0.86(2)	1.98(3)	2.789(5)	155(5)
O24*C*H24*A*O33	0.86(2)	1.91(4)	2.78(3)	179(5)

Symmetry codes: (i) 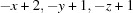; (ii) 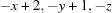; (iii) 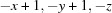; (iv) 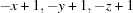.

**Table 2:** Structural feature comparison () of Y^III^Na(OTMA)_4_[12-_MCMn(III)N(shi)_-4](H_2_O)_3.76_(DMF)_0.24_8.04DMF0.62H_2_O (**1**) and Y^III^Na(OAc)_4_[12-MC_Mn(III)N(shi)_-4](H_2_O)_4_6DMF (**2**)

Compound	Y^III^ crystal radius	MC cavity radius	Avg. adjacent Mn^III^Mn^III^ distance	Avg. cross-cavity Mn^III^Mn^III^ distance	Avg. cross-cavity O_ox_O_ox_ distance	Y^III^O_car_MP distance	Y^III^O_ox_MP distance	Y^III^MnMP distance	Na^I^O_solvent_MP distance	Na^I^O_ox_MP distance
(**1**)	1.05	0.55	4.62	6.53	3.71	1.07	1.57	1.91	0.67	1.97
(**2A**)	1.05	0.55	4.61	6.52	3.70	1.04	1.57	1.92	0.79	1.92
(**2B**)	1.05	0.55	4.61	6.52	3.70	1.03	1.58	1.93	0.79	1.91

**Table 3:** Experimental details

Crystal data	
Chemical formula	[YNaMn_4_(C_7_H_4_NO_3_)_4_(C_5_H_9_O_2_)_4_(C_3_H_7_NO)_0.24_(H_2_O)_3.76_]8.04C_3_H_7_NO0.62H_2_O
*M*_r_	2021.04
Crystal system, space group	Triclinic, *P* 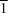
Temperature (K)	100
*a*, *b*, *c* ()	14.8659(9), 17.3261(10), 19.2709(11)
, , ()	83.488(3), 82.499(3), 72.805(3)
*V* (^3^)	4686.5(5)
*Z*	2
Radiation type	Cu *K*
(mm^1^)	5.83
Crystal size (mm)	0.15 0.14 0.10

Data collection	
Diffractometer	Bruker X8 Prospector CCD
Absorption correction	Multi-scan (*SADABS*; Bruker, 2014 ▸)
*T*_min_, *T*_max_	0.572, 0.753
No. of measured, independent and observed [*I* > 2(*I*)] reflections	59383, 16375, 14639
*R*_int_	0.045
(sin /)_max_ (^1^)	0.596

Refinement	
*R*[*F* ^2^ > 2(*F* ^2^)], *wR*(*F* ^2^), *S*	0.053, 0.142, 1.02
No. of reflections	16375
No. of parameters	1537
No. of restraints	1505
H-atom treatment	H atoms treated by a mixture of independent and constrained refinement
_max_, _min_ (e ^3^)	1.73, 0.58

Computer programs: *APEX2* and *SAINT* (Bruker, 2014 ▸), *SHELXS97* (Sheldrick, 2008 ▸), *SHELXL2014* (Sheldrick, 2008 ▸) and *SHELXLE* (Hbschle *et al.*, 2011 ▸), *Mercury* (Macrae *et al.*, 2006 ▸) and *publCIF* (Westrip, 2010 ▸).

## supplementary crystallographic information

### Crystal data

**Table:**

[YNaMn_4_(C_7_H_4_NO_3_)_4_(C_5_H_9_O_2_)_4_(C_3_H_7_NO)\ 0.24(H_2_O)_3.76_]·8.04C_3_H_7_NO·0.62H_2_O	*Z* = 2
*M**_r_* = 2021.04	*F*(000) = 2106.3
Triclinic, *P*1	*D*_x_ = 1.432 Mg m^−^^3^
*a* = 14.8659 (9) Å	Cu *K*α radiation, λ = 1.54178 Å
*b* = 17.3261 (10) Å	Cell parameters from 9921 reflections
*c* = 19.2709 (11) Å	θ = 2.7–66.8°
α = 83.488 (3)°	µ = 5.83 mm^−^^1^
β = 82.499 (3)°	*T* = 100 K
γ = 72.805 (3)°	Plate, black
*V* = 4686.5 (5) Å^3^	0.15 × 0.14 × 0.10 mm

### Data collection

**Table:**

Bruker X8 Prospector CCD diffractometer	16375 independent reflections
Radiation source: I-mu-S microsource X-ray tube	14639 reflections with *I* > 2σ(*I*)
Laterally graded multilayer (Goebel) mirror monochromator	*R*_int_ = 0.045
ω and phi scans	θ_max_ = 66.9°, θ_min_ = 2.3°
Absorption correction: multi-scan (*SADABS*; Bruker, 2014)	*h* = −17→17
*T*_min_ = 0.572, *T*_max_ = 0.753	*k* = −20→20
59383 measured reflections	*l* = −22→22

### Refinement

**Table:**

Refinement on *F*^2^	Primary atom site location: structure-invariant direct methods
Least-squares matrix: full	Secondary atom site location: difference Fourier map
*R*[*F*^2^ > 2σ(*F*^2^)] = 0.053	Hydrogen site location: mixed
*wR*(*F*^2^) = 0.142	H atoms treated by a mixture of independent and constrained refinement
*S* = 1.02	*w* = 1/[σ^2^(*F*_o_^2^) + (0.0737*P*)^2^ + 11.125*P*] where *P* = (*F*_o_^2^ + 2*F*_c_^2^)/3
16375 reflections	(Δ/σ)_max_ = 0.005
1537 parameters	Δρ_max_ = 1.73 e Å^−^^3^
1505 restraints	Δρ_min_ = −0.58 e Å^−^^3^

### Special details

**Table:**

Geometry. All e.s.d.'s (except the e.s.d. in the dihedral angle between two l.s. planes) are estimated using the full covariance matrix. The cell e.s.d.'s are taken into account individually in the estimation of e.s.d.'s in distances, angles and torsion angles; correlations between e.s.d.'s in cell parameters are only used when they are defined by crystal symmetry. An approximate (isotropic) treatment of cell e.s.d.'s is used for estimating e.s.d.'s involving l.s. planes.
Refinement. For all of the disordered solvate water and DMF molecules, neighboring atoms were restrained to have similar *U*^*ij*^ components of their ADPs if closter than 1.7 Angstoms (SIMU restraints in *SHELXL*).The geometries of the DMF molecules associated with N7, N8B, N9, N9B, N10, N10B, N11, N12, N12B, N13, and N13B were restrained to be similar to the DMF molecule associated with N5 (e.s.d. = 0.02 Angstrom). For the DMF molecules associated with N7B and N11B, the geometries were restrained to be similar to the DMF molecule associated with N5 (e.s.d. = 0.001 Angstrom). For the DMF molecules associated with N8B, N11B, and N13B, the carbon, oxygen, and nitrogen atoms were restrained to lie in the same plane (0.01 Angstroms cubed).A water molecule (O24C) and DMF molecule associated with N13 are disordered over a binding site to Na1. The atoms O24 and O24C were given identical coordinates, and to avoid correlation of the thermal parameters, the ADP of O24 and O24C were constrained to be identical. Subject to these and the above conditions, the occupancy ratio of the disordered water and DMF molecules refined to 0.758 (8) to 0.242 (8). Correlated to the occupation of the binding site is a DMF molecule associated with N13B and a DMF molecule associated with N7 that is disordered over two sites with different orientations. Subject to the above restraints, the occupancy ratio of the DMF molecule associated with N13B refined to 0.252 (5), and the occupancy ratio of the disordered DMF molecule associated with N7 refined to 0.748 (5) to 0.252 (5). In addition, two partially occupied water molecules associated with O33 and O34 are correlated to these water and DMF molecules. The occupancy of the water molecule of O33 and the water molecule of O34 are 0.257 (14) and 0.361 (13), respectively.Several DMF molecules are disordered, and the above restraints were used to model the data. The DMF molecule associated with N8 is flipped disordered over two sites, and the occupancy ratio refined to 0.813 (7) to 0.187 (7). The DMF molecule associated with N9 is flipped disordered over two sites, and the occupancy ratio refined to 0.813 (7) to 0.187 (7). The DMF molecule associated with N10 is disordered over two sites with different orientations, and the occupancy ratio refined to 0.795 (6) to 0.205 (6). The DMF molecule associated with N11 is flipped disordered over two sites, and the occupancy ratio refined to 0.790 (9) to 0.210 (9). Two DMF molecules associated with N12 and N12B are partially occupied. The occupancy of the DMF molecule N12 and the DMF molecule 12B are 0.662 (8) and 0.129 (7), respectively.For the water molecules, the oxygen-hydrogen bond distances were restrained to 0.84 (2) Angstrom. The hydrogen-hydrogen distances for the water molecules associated with O24, O33, and O34 were restrained to 1.36 (2) Angstroms. For the water molecule O24C, the hydrogen atoms were restrained to a distance of at least 2.90 (2) Angstroms from Na1. For the water molecules associated with O33 and O34, the hydrogen atoms were refined as riding on the oxygen atoms.For the methyl group carbon atoms 56B, 62B, 63B, 69, 69B, 71B, 72B, 74, 74B, 75, and 75B, hydrogen atoms were placed in tetrahedral positions with an ideal staggered geometry (AFIX 33). All other methyl group hydrogen atoms were allowed to rotate. All other hydrogen atoms were placed in calculated positions and refined as riding on their carrier atoms with C—H distances of 0.95 Angstrom for sp2 carbon atoms and 0.98 Angstrom for methyl carbon atoms. The *U*_iso_ values for hydrogen atoms were set to a multiple of the value of the carrying carbon atom (1.2 times for sp2 hybridized carbon atoms or 1.5 times for methyl carbon atoms and water oxygen atoms).The following low angle reflections were affected by the beam stop and were omitted from the refinement: 1 0 0, 0 1 0, −1 − 1 1, and −1 1 0.

### Fractional atomic coordinates and isotropic or equivalent isotropic displacement parameters (Å^2^)

**Table:**

	*x*	*y*	*z*	*U*_iso_*/*U*_eq_	Occ. (<1)
C1	0.7096 (2)	0.7443 (2)	0.05094 (17)	0.0206 (7)	
C2	0.6469 (3)	0.7852 (2)	−0.00412 (18)	0.0240 (7)	
C3	0.5705 (3)	0.8557 (2)	0.00655 (18)	0.0241 (7)	
C4	0.5182 (3)	0.8919 (2)	−0.05003 (19)	0.0277 (8)	
H4	0.4674	0.9399	−0.0441	0.033*	
C5	0.5393 (3)	0.8590 (3)	−0.1141 (2)	0.0319 (9)	
H5	0.5032	0.8849	−0.1518	0.038*	
C6	0.6124 (3)	0.7886 (3)	−0.1243 (2)	0.0359 (9)	
H6	0.6255	0.7656	−0.1683	0.043*	
C7	0.6660 (3)	0.7522 (2)	−0.0695 (2)	0.0302 (8)	
H7	0.7164	0.7041	−0.0763	0.036*	
C8	0.5234 (2)	0.9402 (2)	0.26837 (18)	0.0212 (7)	
C9	0.4547 (3)	0.9900 (2)	0.31967 (19)	0.0245 (7)	
C10	0.4589 (3)	0.9758 (2)	0.39318 (19)	0.0246 (7)	
C11	0.3913 (3)	1.0274 (2)	0.4371 (2)	0.0289 (8)	
H11	0.3937	1.0185	0.4865	0.035*	
C12	0.3214 (3)	1.0907 (2)	0.4107 (2)	0.0340 (9)	
H12	0.2768	1.1254	0.4418	0.041*	
C13	0.3150 (3)	1.1048 (3)	0.3384 (2)	0.0388 (10)	
H13	0.2660	1.1481	0.3202	0.047*	
C14	0.3812 (3)	1.0545 (2)	0.2938 (2)	0.0330 (9)	
H14	0.3771	1.0636	0.2446	0.040*	
C15	0.7409 (3)	0.7195 (2)	0.45908 (18)	0.0226 (7)	
C16	0.7741 (3)	0.6612 (2)	0.51876 (18)	0.0248 (8)	
C17	0.8440 (3)	0.5863 (2)	0.50983 (19)	0.0258 (8)	
C18	0.8731 (3)	0.5362 (2)	0.5704 (2)	0.0312 (8)	
H18	0.9202	0.4858	0.5656	0.037*	
C19	0.8348 (3)	0.5590 (2)	0.6365 (2)	0.0349 (9)	
H19	0.8564	0.5244	0.6765	0.042*	
C20	0.7645 (3)	0.6322 (3)	0.6456 (2)	0.0374 (10)	
H20	0.7379	0.6473	0.6913	0.045*	
C21	0.7344 (3)	0.6823 (2)	0.5869 (2)	0.0315 (9)	
H21	0.6860	0.7318	0.5927	0.038*	
C22	0.9346 (2)	0.5280 (2)	0.24097 (18)	0.0223 (7)	
C23	0.9801 (3)	0.4625 (2)	0.1944 (2)	0.0255 (8)	
C24	0.9629 (3)	0.4678 (2)	0.12331 (19)	0.0254 (8)	
C25	1.0121 (3)	0.4030 (2)	0.0831 (2)	0.0307 (8)	
H25	1.0020	0.4059	0.0351	0.037*	
C26	1.0744 (3)	0.3355 (2)	0.1116 (2)	0.0357 (9)	
H26	1.1068	0.2924	0.0831	0.043*	
C27	1.0910 (3)	0.3289 (2)	0.1818 (2)	0.0398 (10)	
H27	1.1337	0.2816	0.2014	0.048*	
C28	1.0442 (3)	0.3923 (2)	0.2223 (2)	0.0319 (9)	
H28	1.0555	0.3885	0.2702	0.038*	
C29	0.9785 (3)	0.7356 (2)	0.0956 (2)	0.0275 (8)	
C30	1.0344 (3)	0.7837 (3)	0.0444 (2)	0.0411 (11)	
C31	1.0753 (4)	0.8352 (3)	0.0832 (3)	0.0509 (13)	
H31A	1.1078	0.8671	0.0492	0.076*	
H31B	1.1204	0.8000	0.1142	0.076*	
H31C	1.0240	0.8719	0.1112	0.076*	
C32	1.1134 (4)	0.7254 (4)	0.0017 (3)	0.0678 (18)	
H32A	1.0862	0.6939	−0.0244	0.102*	
H32B	1.1565	0.6886	0.0333	0.102*	
H32C	1.1484	0.7561	−0.0314	0.102*	
C33	0.9635 (5)	0.8392 (3)	−0.0041 (3)	0.0616 (16)	
H33A	0.9130	0.8765	0.0239	0.092*	
H33B	0.9361	0.8061	−0.0283	0.092*	
H33C	0.9962	0.8702	−0.0388	0.092*	
C34	0.7526 (3)	0.9651 (2)	0.12915 (19)	0.0290 (8)	
C35	0.7756 (3)	1.0463 (2)	0.1244 (2)	0.0371 (10)	
C36	0.7445 (4)	1.0949 (3)	0.0567 (3)	0.0551 (13)	
H36A	0.7590	1.1468	0.0535	0.083*	
H36B	0.6762	1.1046	0.0562	0.083*	
H36C	0.7782	1.0645	0.0166	0.083*	
C37	0.7184 (3)	1.0906 (3)	0.1889 (3)	0.0492 (12)	
H37A	0.6511	1.0959	0.1883	0.074*	
H37B	0.7276	1.1446	0.1870	0.074*	
H37C	0.7406	1.0593	0.2322	0.074*	
C38	0.8809 (3)	1.0344 (3)	0.1280 (3)	0.0458 (11)	
H38A	0.9175	1.0059	0.0878	0.069*	
H38B	0.9002	1.0023	0.1717	0.069*	
H38C	0.8925	1.0873	0.1270	0.069*	
C39	0.7880 (3)	0.9357 (2)	0.3575 (2)	0.0286 (8)	
C40	0.8520 (3)	0.9791 (3)	0.3829 (2)	0.0401 (10)	
C41	0.9221 (3)	1.0004 (3)	0.3245 (3)	0.0461 (11)	
H41A	0.9637	0.9507	0.3053	0.069*	
H41B	0.9602	1.0291	0.3434	0.069*	
H41C	0.8876	1.0353	0.2872	0.069*	
C42	0.9077 (4)	0.9190 (4)	0.4386 (3)	0.0657 (17)	
H42A	0.9432	0.8685	0.4175	0.099*	
H42B	0.8635	0.9075	0.4779	0.099*	
H42C	0.9519	0.9430	0.4557	0.099*	
C43	0.7913 (5)	1.0551 (4)	0.4162 (4)	0.0710 (19)	
H43A	0.7548	1.0922	0.3810	0.107*	
H43B	0.8321	1.0816	0.4345	0.107*	
H43C	0.7479	1.0406	0.4548	0.107*	
C44	1.0183 (3)	0.7055 (2)	0.32290 (19)	0.0292 (8)	
C45	1.1210 (3)	0.7093 (3)	0.3051 (2)	0.0350 (9)	
C46	1.1251 (3)	0.7950 (3)	0.2804 (3)	0.0467 (11)	
H46A	1.0880	0.8152	0.2403	0.070*	
H46B	1.1911	0.7944	0.2663	0.070*	
H46C	1.0989	0.8304	0.3187	0.070*	
C47	1.1641 (3)	0.6538 (3)	0.2449 (3)	0.0471 (11)	
H47A	1.1253	0.6716	0.2055	0.071*	
H47B	1.1658	0.5978	0.2615	0.071*	
H47C	1.2286	0.6567	0.2295	0.071*	
C48	1.1755 (4)	0.6780 (3)	0.3692 (3)	0.0542 (13)	
H48A	1.1759	0.6217	0.3828	0.081*	
H48B	1.1449	0.7114	0.4082	0.081*	
H48C	1.2408	0.6808	0.3579	0.081*	
O26	0.3919 (2)	0.8164 (2)	0.29808 (18)	0.0521 (8)	
C52	0.3186 (3)	0.8741 (3)	0.3082 (3)	0.0495 (12)	
H52	0.2980	0.9116	0.2696	0.059*	
N6	0.2689 (3)	0.8853 (3)	0.3696 (2)	0.0510 (10)	
C53	0.2997 (5)	0.8315 (4)	0.4300 (3)	0.0709 (17)	
H53A	0.3443	0.7810	0.4145	0.106*	
H53B	0.2448	0.8196	0.4581	0.106*	
H53C	0.3309	0.8573	0.4585	0.106*	
C54	0.1848 (4)	0.9538 (4)	0.3792 (4)	0.0719 (19)	
H54A	0.1966	0.9910	0.4092	0.108*	
H54B	0.1316	0.9342	0.4013	0.108*	
H54C	0.1695	0.9822	0.3334	0.108*	
O27	0.4821 (5)	0.7058 (5)	0.5099 (3)	0.099 (2)	0.748 (5)
C55	0.4849 (6)	0.7581 (7)	0.5502 (4)	0.082 (2)	0.748 (5)
H55	0.5191	0.7953	0.5308	0.099*	0.748 (5)
N7	0.4468 (6)	0.7670 (6)	0.6144 (4)	0.089 (2)	0.748 (5)
C56	0.3832 (8)	0.7197 (8)	0.6489 (6)	0.123 (4)	0.748 (5)
H56A	0.4048	0.6947	0.6943	0.184*	0.748 (5)
H56B	0.3839	0.6773	0.6190	0.184*	0.748 (5)
H56C	0.3186	0.7556	0.6563	0.184*	0.748 (5)
C57	0.4594 (8)	0.8258 (8)	0.6569 (6)	0.106 (3)	0.748 (5)
H57A	0.4864	0.8647	0.6270	0.127*	0.748 (5)
H57B	0.5023	0.7980	0.6923	0.127*	0.748 (5)
H57C	0.3980	0.8545	0.6804	0.127*	0.748 (5)
O27B	0.486 (2)	0.7133 (14)	0.7576 (9)	0.175 (10)	0.252 (5)
C55B	0.4904 (18)	0.7687 (13)	0.7122 (8)	0.116 (6)	0.252 (5)
H55B	0.5196	0.8075	0.7223	0.140*	0.252 (5)
N7B	0.4572 (12)	0.7783 (8)	0.6510 (7)	0.098 (4)	0.252 (5)
C56B	0.4258 (19)	0.7137 (11)	0.6292 (11)	0.125 (7)	0.252 (5)
H56D	0.4030	0.7296	0.5827	0.188*	0.252 (5)
H56E	0.4787	0.6641	0.6271	0.188*	0.252 (5)
H56F	0.3743	0.7040	0.6631	0.188*	0.252 (5)
C57B	0.4632 (18)	0.8464 (12)	0.6004 (10)	0.096 (5)	0.252 (5)
H57D	0.5255	0.8554	0.5993	0.115*	0.252 (5)
H57E	0.4137	0.8952	0.6142	0.115*	0.252 (5)
H57F	0.4546	0.8342	0.5537	0.115*	0.252 (5)
O28	0.7538 (3)	0.4474 (3)	0.2625 (3)	0.0522 (12)	0.813 (7)
C58	0.8097 (5)	0.3802 (4)	0.2689 (4)	0.0566 (16)	0.813 (7)
H58	0.8491	0.3708	0.3058	0.068*	0.813 (7)
N8	0.8218 (5)	0.3195 (4)	0.2308 (4)	0.0559 (14)	0.813 (7)
C59	0.7602 (8)	0.3316 (6)	0.1739 (5)	0.090 (3)	0.813 (7)
H59A	0.7993	0.3163	0.1299	0.134*	0.813 (7)
H59B	0.7235	0.3888	0.1687	0.134*	0.813 (7)
H59C	0.7170	0.2979	0.1856	0.134*	0.813 (7)
C60	0.8925 (7)	0.2423 (5)	0.2366 (5)	0.078 (2)	0.813 (7)
H60A	0.9309	0.2324	0.1914	0.117*	0.813 (7)
H60B	0.8613	0.1993	0.2497	0.117*	0.813 (7)
H60C	0.9331	0.2424	0.2727	0.117*	0.813 (7)
O28B	0.7439 (16)	0.4344 (12)	0.2246 (14)	0.068 (5)	0.187 (7)
C58B	0.8040 (16)	0.3744 (12)	0.2020 (13)	0.060 (3)	0.187 (7)
H58B	0.8433	0.3831	0.1607	0.072*	0.187 (7)
N8B	0.8185 (17)	0.3000 (13)	0.2296 (14)	0.065 (4)	0.187 (7)
C59B	0.765 (3)	0.276 (2)	0.2927 (17)	0.093 (8)	0.187 (7)
H59D	0.7304	0.3243	0.3178	0.139*	0.187 (7)
H59E	0.8078	0.2377	0.3232	0.139*	0.187 (7)
H59F	0.7191	0.2507	0.2797	0.139*	0.187 (7)
C60B	0.892 (2)	0.2353 (17)	0.198 (2)	0.082 (7)	0.187 (7)
H60D	0.9536	0.2436	0.1996	0.123*	0.187 (7)
H60E	0.8811	0.2349	0.1484	0.123*	0.187 (7)
H60F	0.8898	0.1833	0.2228	0.123*	0.187 (7)
O24B	0.5766 (16)	0.6104 (12)	0.4575 (11)	0.094 (5)	0.252 (5)
C73B	0.6222 (16)	0.5394 (13)	0.4589 (17)	0.093 (5)	0.252 (5)
H73B	0.6528	0.5246	0.4139	0.112*	0.252 (5)
N13B	0.6396 (13)	0.4791 (12)	0.5058 (12)	0.101 (4)	0.252 (5)
C74B	0.605 (3)	0.480 (2)	0.5799 (14)	0.142 (11)	0.252 (5)
H74D	0.6289	0.4257	0.6033	0.213*	0.252 (5)
H74E	0.6259	0.5190	0.6015	0.213*	0.252 (5)
H74F	0.5352	0.4953	0.5848	0.213*	0.252 (5)
C75B	0.700 (2)	0.4037 (16)	0.4804 (17)	0.101 (6)	0.252 (5)
H75D	0.7097	0.3615	0.5194	0.152*	0.252 (5)
H75E	0.6709	0.3875	0.4441	0.152*	0.252 (5)
H75F	0.7617	0.4111	0.4606	0.152*	0.252 (5)
O29	0.6538 (5)	0.4705 (4)	0.0592 (3)	0.094 (2)	0.813 (7)
C61	0.7226 (5)	0.4828 (4)	0.0228 (4)	0.0627 (18)	0.813 (7)
H61	0.7729	0.4889	0.0455	0.075*	0.813 (7)
N9	0.7307 (5)	0.4879 (4)	−0.0460 (3)	0.0588 (15)	0.813 (7)
C62	0.6558 (7)	0.4829 (6)	−0.0858 (5)	0.085 (3)	0.813 (7)
H62A	0.6515	0.5217	−0.1272	0.127*	0.813 (7)
H62B	0.5953	0.4956	−0.0562	0.127*	0.813 (7)
H62C	0.6703	0.4279	−0.1007	0.127*	0.813 (7)
C63	0.8174 (6)	0.4974 (5)	−0.0839 (6)	0.091 (3)	0.813 (7)
H63A	0.8511	0.4480	−0.1072	0.137*	0.813 (7)
H63B	0.8573	0.5071	−0.0510	0.137*	0.813 (7)
H63C	0.8029	0.5436	−0.1191	0.137*	0.813 (7)
O29B	0.593 (2)	0.474 (2)	−0.0371 (18)	0.112 (7)	0.187 (7)
C61B	0.670 (2)	0.460 (2)	−0.075 (2)	0.072 (4)	0.187 (7)
H61B	0.6843	0.4166	−0.1042	0.087*	0.187 (7)
N9B	0.732 (2)	0.501 (2)	−0.0781 (14)	0.073 (4)	0.187 (7)
C62B	0.783 (3)	0.505 (2)	−0.0198 (18)	0.085 (6)	0.187 (7)
H62D	0.8250	0.5392	−0.0348	0.128*	0.187 (7)
H62E	0.7376	0.5281	0.0194	0.128*	0.187 (7)
H62F	0.8203	0.4503	−0.0048	0.128*	0.187 (7)
C63B	0.770 (3)	0.529 (2)	−0.1455 (16)	0.096 (8)	0.187 (7)
H63D	0.8149	0.5587	−0.1388	0.144*	0.187 (7)
H63E	0.8024	0.4829	−0.1729	0.144*	0.187 (7)
H63F	0.7183	0.5657	−0.1707	0.144*	0.187 (7)
O30	0.1094 (4)	1.1136 (3)	0.2412 (3)	0.0700 (15)	0.795 (6)
C64	0.1762 (5)	1.1008 (3)	0.1954 (3)	0.0485 (15)	0.795 (6)
H64	0.2132	1.1378	0.1889	0.058*	0.795 (6)
N10	0.2021 (4)	1.0394 (3)	0.1536 (3)	0.0428 (12)	0.795 (6)
C65	0.1457 (6)	0.9836 (6)	0.1555 (5)	0.053 (2)	0.795 (6)
H65A	0.0982	0.9918	0.1964	0.064*	0.795 (6)
H65B	0.1869	0.9278	0.1587	0.064*	0.795 (6)
H65C	0.1138	0.9935	0.1125	0.064*	0.795 (6)
C66	0.2827 (6)	1.0294 (5)	0.1004 (5)	0.060 (2)	0.795 (6)
H66A	0.2614	1.0314	0.0540	0.091*	0.795 (6)
H66B	0.3290	0.9769	0.1101	0.091*	0.795 (6)
H66C	0.3120	1.0730	0.1013	0.091*	0.795 (6)
O30B	0.315 (2)	0.8658 (15)	0.1790 (17)	0.128 (9)	0.205 (6)
C64B	0.3147 (19)	0.9280 (15)	0.1396 (17)	0.080 (5)	0.205 (6)
H64B	0.3701	0.9276	0.1087	0.096*	0.205 (6)
N10B	0.2436 (15)	0.9936 (12)	0.1381 (13)	0.063 (4)	0.205 (6)
C65B	0.149 (2)	0.996 (3)	0.173 (2)	0.061 (7)	0.205 (6)
H65D	0.1260	1.0420	0.2013	0.073*	0.205 (6)
H65E	0.1528	0.9454	0.2025	0.073*	0.205 (6)
H65F	0.1062	1.0008	0.1368	0.073*	0.205 (6)
C66B	0.253 (2)	1.0673 (16)	0.099 (2)	0.068 (6)	0.205 (6)
H66D	0.2186	1.0773	0.0574	0.102*	0.205 (6)
H66E	0.3202	1.0620	0.0844	0.102*	0.205 (6)
H66F	0.2271	1.1128	0.1287	0.102*	0.205 (6)
O32	0.4946 (10)	0.3953 (7)	0.2133 (6)	0.149 (4)	0.662 (8)
C70	0.5017 (10)	0.4644 (8)	0.2209 (7)	0.104 (3)	0.662 (8)
H70	0.5351	0.4896	0.1843	0.125*	0.662 (8)
N12	0.4647 (8)	0.5032 (6)	0.2776 (6)	0.097 (3)	0.662 (8)
C71	0.4145 (10)	0.4772 (9)	0.3425 (7)	0.119 (4)	0.662 (8)
H71A	0.3821	0.5243	0.3692	0.178*	0.662 (8)
H71B	0.4600	0.4378	0.3708	0.178*	0.662 (8)
H71C	0.3679	0.4521	0.3308	0.178*	0.662 (8)
C72	0.4817 (9)	0.5813 (7)	0.2843 (8)	0.104 (4)	0.662 (8)
H72A	0.5500	0.5745	0.2792	0.156*	0.662 (8)
H72B	0.4542	0.6002	0.3305	0.156*	0.662 (8)
H72C	0.4522	0.6212	0.2476	0.156*	0.662 (8)
O32B	0.393 (4)	0.438 (3)	0.258 (3)	0.125 (6)	0.129 (7)
C70B	0.383 (3)	0.489 (4)	0.302 (3)	0.102 (5)	0.129 (7)
H70B	0.3305	0.4985	0.3364	0.123*	0.129 (7)
N12B	0.447 (4)	0.529 (4)	0.299 (2)	0.102 (4)	0.129 (7)
C71B	0.501 (4)	0.539 (4)	0.231 (2)	0.104 (7)	0.129 (7)
H71D	0.5457	0.5696	0.2364	0.156*	0.129 (7)
H71E	0.5366	0.4855	0.2158	0.156*	0.129 (7)
H71F	0.4580	0.5684	0.1968	0.156*	0.129 (7)
C72B	0.485 (5)	0.540 (4)	0.362 (3)	0.113 (7)	0.129 (7)
H72D	0.5305	0.5710	0.3489	0.170*	0.129 (7)
H72E	0.4334	0.5684	0.3947	0.170*	0.129 (7)
H72F	0.5167	0.4863	0.3836	0.170*	0.129 (7)
N1	0.7053 (2)	0.78168 (17)	0.10739 (14)	0.0199 (6)	
N2	0.6028 (2)	0.89077 (17)	0.28938 (14)	0.0204 (6)	
N3	0.7847 (2)	0.70711 (17)	0.39560 (14)	0.0206 (6)	
N4	0.8901 (2)	0.60026 (17)	0.21382 (15)	0.0203 (6)	
O1	0.76938 (17)	0.73567 (14)	0.15509 (12)	0.0214 (5)	
O2	0.76892 (17)	0.67437 (14)	0.04144 (12)	0.0239 (5)	
O3	0.54324 (18)	0.88879 (15)	0.06758 (13)	0.0281 (6)	
O4	0.66176 (17)	0.84794 (15)	0.23461 (12)	0.0221 (5)	
O5	0.50616 (17)	0.94708 (15)	0.20380 (12)	0.0244 (5)	
O6	0.52302 (18)	0.91461 (16)	0.42318 (13)	0.0292 (6)	
O7	0.74262 (17)	0.76747 (14)	0.34465 (12)	0.0212 (5)	
O8	0.67041 (18)	0.78276 (15)	0.47023 (12)	0.0252 (5)	
O9	0.88376 (19)	0.55938 (16)	0.44801 (13)	0.0314 (6)	
O10	0.85166 (17)	0.65657 (14)	0.26463 (12)	0.0206 (5)	
O11	0.94163 (18)	0.51425 (15)	0.30769 (12)	0.0259 (5)	
O12	0.90092 (19)	0.53000 (15)	0.09247 (13)	0.0284 (6)	
O13	0.95902 (18)	0.75362 (15)	0.15842 (13)	0.0259 (5)	
O14	0.95353 (18)	0.68252 (15)	0.07062 (13)	0.0277 (6)	
O15	0.81358 (18)	0.90155 (15)	0.14887 (13)	0.0277 (6)	
O16	0.67229 (19)	0.96743 (16)	0.11445 (14)	0.0303 (6)	
O17	0.80583 (19)	0.91298 (15)	0.29535 (13)	0.0296 (6)	
O18	0.71982 (19)	0.92547 (16)	0.39915 (13)	0.0295 (6)	
O19	0.95190 (18)	0.76711 (15)	0.30493 (13)	0.0274 (6)	
O20	1.00531 (19)	0.64178 (16)	0.35380 (14)	0.0300 (6)	
O21	0.7043 (2)	0.59229 (17)	0.17790 (15)	0.0327 (6)	
H21A	0.664 (3)	0.599 (3)	0.150 (2)	0.049*	
H21B	0.715 (4)	0.5427 (13)	0.189 (3)	0.049*	
O22	0.5360 (2)	0.76725 (18)	0.19837 (15)	0.0337 (6)	
H22A	0.518 (4)	0.742 (3)	0.171 (2)	0.050*	
H22B	0.482 (2)	0.778 (3)	0.219 (3)	0.050*	
O23	0.5523 (2)	0.74493 (19)	0.36839 (16)	0.0385 (7)	
H23A	0.534 (4)	0.725 (3)	0.4070 (17)	0.058*	
H23B	0.4944 (19)	0.766 (3)	0.359 (3)	0.058*	
O24C	0.7301 (2)	0.57216 (17)	0.34800 (16)	0.0374 (7)	0.758 (8)
H24A	0.693 (3)	0.566 (2)	0.3854 (16)	0.056*	0.758 (8)
H24B	0.742 (4)	0.5272 (10)	0.3291 (16)	0.056*	0.758 (8)
O24	0.7301 (2)	0.57216 (17)	0.34800 (16)	0.0374 (7)	0.242 (8)
C73	0.7336 (14)	0.5073 (10)	0.3849 (10)	0.053 (3)	0.242 (8)
H73	0.7839	0.4622	0.3699	0.064*	0.242 (8)
N13	0.6797 (15)	0.4908 (12)	0.4407 (11)	0.083 (4)	0.242 (8)
C74	0.589 (2)	0.554 (2)	0.455 (2)	0.086 (6)	0.242 (8)
H74A	0.5533	0.5370	0.4973	0.129*	0.242 (8)
H74B	0.6016	0.6050	0.4618	0.129*	0.242 (8)
H74C	0.5515	0.5626	0.4149	0.129*	0.242 (8)
C75	0.672 (3)	0.4096 (14)	0.458 (2)	0.105 (7)	0.242 (8)
H75A	0.6286	0.4087	0.5014	0.157*	0.242 (8)
H75B	0.6465	0.3926	0.4199	0.157*	0.242 (8)
H75C	0.7341	0.3723	0.4658	0.157*	0.242 (8)
O33	0.614 (2)	0.5511 (17)	0.4696 (16)	0.097 (4)	0.257 (14)
H33E	0.6474	0.5763	0.4848	0.145*	0.257 (14)
H33F	0.5717	0.5468	0.5014	0.145*	0.257 (14)
O34	0.6567 (9)	0.3773 (8)	0.5029 (7)	0.073 (4)	0.361 (13)
H34A	0.6407	0.4059	0.5379	0.109*	0.361 (13)
H34B	0.6056	0.3767	0.4882	0.109*	0.361 (13)
O25	0.5452 (2)	0.6557 (2)	0.10663 (18)	0.0504 (8)	
C49	0.5246 (4)	0.6532 (3)	0.0472 (3)	0.0533 (12)	
H49	0.5702	0.6184	0.0167	0.064*	
N5	0.4446 (3)	0.6948 (3)	0.0228 (2)	0.0572 (11)	
C50	0.3745 (4)	0.7508 (5)	0.0669 (3)	0.087 (2)	
H50A	0.3800	0.8058	0.0546	0.130*	
H50B	0.3852	0.7350	0.1163	0.130*	
H50C	0.3110	0.7494	0.0596	0.130*	
C51	0.4265 (5)	0.6905 (4)	−0.0491 (3)	0.0772 (19)	
H51A	0.4089	0.7452	−0.0727	0.116*	
H51B	0.3748	0.6660	−0.0485	0.116*	
H51C	0.4838	0.6573	−0.0745	0.116*	
O31	0.8265 (6)	0.2547 (5)	0.4425 (5)	0.124 (3)	0.790 (9)
C67	0.9164 (7)	0.2283 (6)	0.4274 (5)	0.086 (2)	0.790 (9)
H67	0.9419	0.1729	0.4181	0.104*	0.790 (9)
N11	0.9754 (5)	0.2716 (5)	0.4240 (4)	0.0768 (19)	0.790 (9)
C68	0.9530 (8)	0.3518 (6)	0.4479 (6)	0.089 (3)	0.790 (9)
H68A	0.9751	0.3877	0.4109	0.133*	0.790 (9)
H68B	0.9844	0.3492	0.4901	0.133*	0.790 (9)
H68C	0.8844	0.3729	0.4588	0.133*	0.790 (9)
C69	1.0730 (6)	0.2372 (6)	0.3946 (6)	0.093 (3)	0.790 (9)
H69A	1.1089	0.2762	0.3953	0.139*	0.790 (9)
H69B	1.1020	0.1872	0.4226	0.139*	0.790 (9)
H69C	1.0737	0.2249	0.3460	0.139*	0.790 (9)
O31B	0.9091 (16)	0.1627 (9)	0.4682 (14)	0.123 (6)	0.210 (9)
C67B	0.8859 (10)	0.2372 (8)	0.4658 (12)	0.092 (4)	0.210 (9)
H67B	0.8207	0.2645	0.4760	0.110*	0.210 (9)
N11B	0.9444 (10)	0.2819 (8)	0.4504 (12)	0.091 (4)	0.210 (9)
C68B	1.0447 (11)	0.2424 (14)	0.435 (2)	0.100 (7)	0.210 (9)
H68D	1.0534	0.1878	0.4207	0.150*	0.210 (9)
H68E	1.0773	0.2388	0.4765	0.150*	0.210 (9)
H68F	1.0710	0.2740	0.3962	0.150*	0.210 (9)
C69B	0.9116 (19)	0.3701 (8)	0.449 (2)	0.098 (7)	0.210 (9)
H69D	0.9656	0.3922	0.4361	0.147*	0.210 (9)
H69E	0.8661	0.3911	0.4137	0.147*	0.210 (9)
H69F	0.8808	0.3864	0.4950	0.147*	0.210 (9)
Na1	0.66419 (10)	0.68887 (9)	0.26918 (8)	0.0300 (3)	
Mn1	0.84339 (4)	0.63331 (3)	0.12120 (3)	0.01926 (13)	
Mn2	0.60623 (4)	0.87295 (3)	0.14702 (3)	0.01915 (13)	
Mn3	0.63617 (4)	0.84893 (3)	0.38408 (3)	0.02020 (13)	
Mn4	0.87530 (4)	0.60976 (3)	0.35842 (3)	0.02184 (14)	
Y1	0.83176 (2)	0.80033 (2)	0.23623 (2)	0.01941 (9)	

### Atomic displacement parameters (Å^2^)

**Table:**

	*U*^11^	*U*^22^	*U*^33^	*U*^12^	*U*^13^	*U*^23^
C1	0.0219 (17)	0.0207 (17)	0.0181 (16)	−0.0060 (14)	−0.0003 (13)	0.0003 (13)
C2	0.0255 (18)	0.0303 (19)	0.0174 (17)	−0.0098 (15)	−0.0057 (14)	0.0018 (14)
C3	0.0284 (19)	0.0244 (18)	0.0206 (17)	−0.0095 (15)	−0.0038 (14)	0.0006 (14)
C4	0.0278 (19)	0.0282 (19)	0.0253 (19)	−0.0062 (16)	−0.0061 (15)	0.0039 (15)
C5	0.032 (2)	0.041 (2)	0.0209 (18)	−0.0080 (18)	−0.0116 (16)	0.0074 (16)
C6	0.042 (2)	0.046 (2)	0.0195 (18)	−0.010 (2)	−0.0057 (17)	−0.0055 (17)
C7	0.030 (2)	0.033 (2)	0.0254 (19)	−0.0042 (16)	−0.0035 (16)	−0.0047 (16)
C8	0.0224 (17)	0.0175 (16)	0.0205 (17)	−0.0027 (14)	−0.0013 (14)	0.0027 (13)
C9	0.0219 (18)	0.0208 (17)	0.0249 (18)	0.0022 (14)	0.0018 (14)	−0.0048 (14)
C10	0.0212 (18)	0.0238 (18)	0.0268 (18)	−0.0026 (15)	−0.0039 (14)	−0.0020 (15)
C11	0.028 (2)	0.032 (2)	0.0239 (18)	−0.0050 (16)	−0.0003 (15)	−0.0042 (15)
C12	0.033 (2)	0.029 (2)	0.032 (2)	0.0035 (17)	0.0058 (17)	−0.0097 (17)
C13	0.036 (2)	0.031 (2)	0.036 (2)	0.0115 (18)	−0.0021 (18)	−0.0039 (18)
C14	0.036 (2)	0.030 (2)	0.0242 (19)	0.0041 (17)	−0.0035 (16)	−0.0010 (16)
C15	0.0255 (18)	0.0220 (17)	0.0204 (17)	−0.0069 (15)	−0.0025 (14)	−0.0018 (14)
C16	0.0308 (19)	0.0275 (19)	0.0162 (17)	−0.0098 (16)	−0.0041 (14)	0.0034 (14)
C17	0.0292 (19)	0.0273 (19)	0.0206 (18)	−0.0087 (15)	−0.0029 (15)	0.0012 (14)
C18	0.037 (2)	0.0269 (19)	0.0268 (19)	−0.0052 (17)	−0.0076 (16)	0.0051 (16)
C19	0.048 (3)	0.034 (2)	0.0216 (19)	−0.0111 (19)	−0.0101 (17)	0.0105 (16)
C20	0.051 (3)	0.039 (2)	0.0174 (18)	−0.008 (2)	−0.0013 (17)	0.0017 (16)
C21	0.039 (2)	0.028 (2)	0.0230 (19)	−0.0044 (17)	−0.0024 (16)	0.0002 (15)
C22	0.0214 (17)	0.0188 (17)	0.0236 (18)	−0.0016 (14)	−0.0023 (14)	0.0001 (14)
C23	0.0251 (18)	0.0180 (17)	0.0293 (19)	0.0007 (14)	−0.0006 (15)	−0.0056 (14)
C24	0.0239 (18)	0.0234 (18)	0.0272 (18)	−0.0045 (15)	−0.0021 (15)	−0.0016 (15)
C25	0.034 (2)	0.0271 (19)	0.030 (2)	−0.0048 (16)	−0.0002 (16)	−0.0096 (16)
C26	0.036 (2)	0.027 (2)	0.041 (2)	0.0009 (17)	−0.0035 (18)	−0.0173 (18)
C27	0.040 (2)	0.023 (2)	0.048 (3)	0.0074 (18)	−0.009 (2)	−0.0071 (18)
C28	0.039 (2)	0.0261 (19)	0.0263 (19)	−0.0001 (17)	−0.0062 (17)	−0.0055 (16)
C29	0.0265 (19)	0.0259 (19)	0.0265 (19)	−0.0033 (15)	0.0034 (15)	−0.0052 (15)
C30	0.050 (3)	0.046 (3)	0.033 (2)	−0.027 (2)	0.016 (2)	−0.0150 (19)
C31	0.062 (3)	0.054 (3)	0.046 (3)	−0.036 (3)	0.025 (2)	−0.024 (2)
C32	0.070 (4)	0.076 (4)	0.071 (4)	−0.051 (3)	0.045 (3)	−0.045 (3)
C33	0.106 (5)	0.055 (3)	0.033 (3)	−0.045 (3)	0.002 (3)	0.006 (2)
C34	0.032 (2)	0.0258 (19)	0.0226 (18)	−0.0020 (16)	0.0029 (15)	0.0021 (15)
C35	0.032 (2)	0.027 (2)	0.051 (3)	−0.0097 (17)	−0.0041 (19)	0.0052 (18)
C36	0.050 (3)	0.043 (3)	0.073 (4)	−0.022 (2)	−0.010 (3)	0.019 (3)
C37	0.041 (3)	0.033 (2)	0.075 (4)	−0.009 (2)	−0.005 (2)	−0.012 (2)
C38	0.037 (2)	0.039 (2)	0.063 (3)	−0.016 (2)	−0.003 (2)	0.004 (2)
C39	0.032 (2)	0.0227 (18)	0.028 (2)	−0.0026 (16)	−0.0017 (16)	−0.0037 (15)
C40	0.045 (3)	0.048 (3)	0.034 (2)	−0.023 (2)	0.0065 (19)	−0.0156 (19)
C41	0.046 (3)	0.051 (3)	0.050 (3)	−0.026 (2)	0.006 (2)	−0.015 (2)
C42	0.061 (3)	0.112 (5)	0.039 (3)	−0.049 (4)	−0.010 (2)	0.001 (3)
C43	0.072 (4)	0.071 (4)	0.084 (4)	−0.044 (3)	0.029 (3)	−0.047 (3)
C44	0.031 (2)	0.032 (2)	0.0231 (18)	−0.0049 (17)	−0.0065 (15)	−0.0053 (16)
C45	0.028 (2)	0.035 (2)	0.042 (2)	−0.0083 (17)	−0.0079 (18)	−0.0016 (18)
C46	0.038 (2)	0.043 (3)	0.062 (3)	−0.017 (2)	−0.007 (2)	−0.004 (2)
C47	0.038 (3)	0.040 (2)	0.058 (3)	−0.010 (2)	0.009 (2)	−0.005 (2)
C48	0.040 (3)	0.064 (3)	0.060 (3)	−0.016 (2)	−0.020 (2)	0.008 (3)
O26	0.0374 (18)	0.068 (2)	0.051 (2)	−0.0147 (17)	0.0045 (15)	−0.0159 (17)
C52	0.032 (2)	0.068 (3)	0.054 (3)	−0.019 (2)	0.001 (2)	−0.021 (3)
N6	0.038 (2)	0.065 (3)	0.056 (3)	−0.022 (2)	0.0059 (19)	−0.025 (2)
C53	0.082 (4)	0.074 (4)	0.063 (4)	−0.038 (4)	0.021 (3)	−0.021 (3)
C54	0.040 (3)	0.086 (4)	0.094 (5)	−0.013 (3)	0.005 (3)	−0.049 (4)
O27	0.104 (5)	0.158 (7)	0.057 (4)	−0.082 (5)	0.000 (3)	0.004 (4)
C55	0.067 (4)	0.142 (6)	0.053 (4)	−0.057 (4)	0.002 (3)	−0.010 (4)
N7	0.072 (4)	0.138 (6)	0.063 (4)	−0.049 (4)	0.008 (3)	0.002 (4)
C56	0.091 (7)	0.172 (9)	0.088 (7)	−0.045 (7)	0.037 (6)	0.031 (7)
C57	0.069 (5)	0.168 (9)	0.083 (6)	−0.035 (6)	0.009 (5)	−0.033 (6)
O27B	0.155 (18)	0.20 (2)	0.144 (18)	−0.016 (18)	−0.013 (16)	−0.023 (17)
C55B	0.094 (9)	0.164 (11)	0.085 (9)	−0.031 (10)	0.005 (9)	−0.014 (9)
N7B	0.079 (6)	0.148 (7)	0.073 (6)	−0.042 (6)	0.000 (6)	−0.010 (6)
C56B	0.097 (11)	0.166 (11)	0.096 (10)	−0.030 (11)	0.016 (10)	0.011 (10)
C57B	0.062 (9)	0.147 (11)	0.088 (10)	−0.051 (9)	0.004 (9)	−0.003 (10)
O28	0.067 (3)	0.032 (2)	0.057 (3)	−0.014 (2)	−0.005 (2)	−0.002 (2)
C58	0.071 (4)	0.038 (3)	0.063 (3)	−0.019 (3)	−0.007 (3)	−0.006 (3)
N8	0.070 (3)	0.039 (3)	0.065 (3)	−0.025 (2)	0.002 (3)	−0.018 (2)
C59	0.121 (7)	0.078 (5)	0.066 (5)	−0.016 (5)	−0.011 (5)	−0.023 (4)
C60	0.084 (5)	0.048 (4)	0.102 (6)	−0.016 (4)	−0.002 (5)	−0.022 (4)
O28B	0.088 (9)	0.047 (8)	0.072 (9)	−0.024 (7)	−0.005 (9)	−0.008 (8)
C58B	0.075 (6)	0.044 (5)	0.066 (6)	−0.025 (5)	−0.004 (5)	−0.010 (5)
N8B	0.078 (6)	0.047 (6)	0.071 (5)	−0.020 (5)	−0.001 (5)	−0.011 (5)
C59B	0.104 (14)	0.073 (13)	0.088 (13)	−0.018 (12)	0.013 (13)	−0.002 (12)
C60B	0.089 (11)	0.060 (11)	0.097 (12)	−0.025 (10)	0.004 (12)	−0.015 (11)
O24B	0.121 (11)	0.086 (10)	0.077 (9)	−0.044 (9)	−0.003 (9)	0.014 (9)
C73B	0.097 (8)	0.096 (7)	0.081 (8)	−0.041 (7)	0.019 (7)	0.017 (7)
N13B	0.099 (7)	0.107 (7)	0.090 (7)	−0.033 (6)	−0.001 (6)	0.018 (6)
C74B	0.113 (18)	0.152 (19)	0.138 (19)	−0.003 (17)	−0.020 (17)	−0.001 (18)
C75B	0.093 (11)	0.119 (11)	0.088 (11)	−0.036 (10)	−0.020 (10)	0.033 (10)
O29	0.122 (5)	0.080 (4)	0.072 (4)	−0.020 (4)	−0.003 (4)	0.002 (3)
C61	0.066 (4)	0.052 (3)	0.073 (4)	−0.014 (3)	−0.016 (3)	−0.016 (3)
N9	0.065 (3)	0.050 (3)	0.068 (4)	−0.027 (2)	0.000 (3)	−0.013 (3)
C62	0.101 (6)	0.078 (6)	0.078 (6)	−0.024 (5)	−0.024 (5)	−0.010 (5)
C63	0.081 (5)	0.067 (5)	0.126 (7)	−0.037 (4)	0.026 (5)	−0.013 (5)
O29B	0.116 (13)	0.098 (13)	0.123 (14)	−0.035 (12)	−0.002 (12)	−0.012 (12)
C61B	0.079 (7)	0.062 (7)	0.083 (7)	−0.028 (7)	−0.008 (7)	−0.011 (7)
N9B	0.077 (6)	0.060 (6)	0.086 (6)	−0.024 (5)	−0.009 (6)	−0.015 (6)
C62B	0.084 (10)	0.067 (10)	0.102 (10)	−0.018 (9)	−0.007 (10)	−0.011 (10)
C63B	0.092 (13)	0.076 (13)	0.120 (14)	−0.032 (12)	0.013 (13)	−0.019 (13)
O30	0.066 (3)	0.068 (3)	0.068 (3)	−0.003 (3)	−0.003 (3)	−0.021 (3)
C64	0.057 (4)	0.038 (3)	0.055 (4)	−0.016 (3)	−0.020 (3)	0.000 (3)
N10	0.049 (3)	0.040 (3)	0.048 (3)	−0.025 (2)	−0.011 (2)	−0.001 (2)
C65	0.061 (4)	0.056 (5)	0.057 (5)	−0.036 (3)	−0.009 (3)	−0.010 (4)
C66	0.068 (5)	0.063 (5)	0.060 (4)	−0.035 (4)	−0.001 (4)	−0.007 (4)
O30B	0.116 (16)	0.104 (16)	0.139 (18)	−0.010 (14)	0.035 (15)	−0.011 (15)
C64B	0.079 (9)	0.078 (9)	0.078 (9)	−0.023 (8)	0.008 (9)	−0.001 (9)
N10B	0.068 (7)	0.064 (7)	0.064 (7)	−0.031 (6)	−0.002 (6)	−0.001 (6)
C65B	0.068 (11)	0.056 (11)	0.065 (12)	−0.026 (10)	−0.003 (10)	−0.015 (10)
C66B	0.072 (11)	0.068 (12)	0.065 (10)	−0.030 (10)	−0.006 (10)	0.013 (11)
O32	0.180 (10)	0.154 (9)	0.113 (7)	−0.032 (8)	−0.060 (7)	0.006 (7)
C70	0.114 (7)	0.106 (7)	0.109 (7)	−0.050 (6)	−0.043 (6)	0.014 (6)
N12	0.087 (5)	0.100 (6)	0.115 (6)	−0.053 (5)	−0.015 (5)	0.026 (5)
C71	0.107 (8)	0.116 (8)	0.130 (9)	−0.049 (7)	−0.002 (7)	0.042 (7)
C72	0.094 (7)	0.098 (7)	0.129 (9)	−0.055 (6)	−0.002 (7)	0.018 (7)
O32B	0.123 (10)	0.127 (10)	0.126 (10)	−0.045 (10)	−0.020 (10)	0.025 (10)
C70B	0.094 (8)	0.104 (9)	0.119 (9)	−0.055 (8)	−0.019 (8)	0.027 (8)
N12B	0.097 (7)	0.103 (8)	0.118 (8)	−0.054 (7)	−0.024 (7)	0.026 (7)
C71B	0.101 (11)	0.103 (11)	0.117 (12)	−0.053 (11)	−0.020 (11)	0.025 (11)
C72B	0.103 (11)	0.110 (11)	0.128 (12)	−0.048 (11)	−0.011 (11)	0.029 (11)
N1	0.0207 (14)	0.0223 (14)	0.0152 (13)	−0.0033 (12)	−0.0052 (11)	0.0015 (11)
N2	0.0187 (14)	0.0221 (14)	0.0166 (14)	−0.0011 (12)	0.0032 (11)	−0.0049 (11)
N3	0.0258 (15)	0.0182 (14)	0.0143 (13)	−0.0022 (12)	−0.0036 (11)	0.0034 (11)
N4	0.0233 (15)	0.0165 (14)	0.0184 (14)	−0.0014 (11)	0.0005 (11)	−0.0054 (11)
O1	0.0227 (12)	0.0190 (11)	0.0187 (11)	0.0004 (10)	−0.0047 (9)	0.0000 (9)
O2	0.0271 (13)	0.0220 (12)	0.0192 (12)	−0.0005 (10)	−0.0028 (10)	−0.0048 (10)
O3	0.0286 (14)	0.0257 (13)	0.0247 (13)	0.0023 (11)	−0.0066 (11)	−0.0033 (10)
O4	0.0200 (12)	0.0258 (12)	0.0155 (11)	0.0008 (10)	0.0007 (9)	−0.0041 (9)
O5	0.0258 (13)	0.0237 (12)	0.0183 (12)	0.0015 (10)	−0.0030 (10)	−0.0011 (10)
O6	0.0281 (14)	0.0337 (14)	0.0199 (12)	−0.0012 (11)	0.0007 (10)	−0.0023 (11)
O7	0.0229 (12)	0.0192 (11)	0.0160 (11)	0.0004 (10)	−0.0012 (9)	0.0031 (9)
O8	0.0286 (13)	0.0238 (13)	0.0175 (12)	0.0004 (11)	0.0002 (10)	−0.0022 (10)
O9	0.0361 (15)	0.0253 (13)	0.0231 (13)	0.0032 (11)	−0.0007 (11)	0.0028 (10)
O10	0.0256 (12)	0.0157 (11)	0.0169 (11)	−0.0005 (9)	0.0000 (9)	−0.0033 (9)
O11	0.0317 (14)	0.0206 (12)	0.0196 (12)	0.0016 (10)	−0.0043 (10)	0.0002 (10)
O12	0.0357 (15)	0.0243 (13)	0.0225 (13)	−0.0022 (11)	−0.0049 (11)	−0.0056 (10)
O13	0.0248 (13)	0.0289 (13)	0.0217 (13)	−0.0053 (11)	0.0026 (10)	−0.0052 (10)
O14	0.0317 (14)	0.0274 (13)	0.0239 (13)	−0.0101 (11)	0.0043 (11)	−0.0052 (11)
O15	0.0290 (14)	0.0254 (13)	0.0247 (13)	−0.0045 (11)	0.0003 (11)	0.0023 (10)
O16	0.0322 (15)	0.0269 (13)	0.0327 (14)	−0.0108 (11)	−0.0058 (11)	0.0036 (11)
O17	0.0367 (15)	0.0253 (13)	0.0258 (13)	−0.0081 (11)	0.0005 (11)	−0.0047 (11)
O18	0.0293 (14)	0.0326 (14)	0.0270 (13)	−0.0101 (11)	0.0041 (11)	−0.0093 (11)
O19	0.0278 (14)	0.0272 (13)	0.0255 (13)	−0.0034 (11)	−0.0066 (11)	−0.0030 (10)
O20	0.0293 (14)	0.0296 (14)	0.0297 (14)	−0.0068 (11)	−0.0073 (11)	0.0044 (11)
O21	0.0360 (16)	0.0293 (14)	0.0341 (15)	−0.0104 (13)	−0.0069 (12)	−0.0006 (12)
O22	0.0302 (15)	0.0360 (16)	0.0348 (16)	−0.0112 (13)	0.0029 (12)	−0.0051 (12)
O23	0.0369 (16)	0.0433 (17)	0.0347 (16)	−0.0144 (14)	0.0015 (13)	0.0018 (13)
O24C	0.0448 (17)	0.0280 (14)	0.0382 (16)	−0.0118 (13)	−0.0002 (13)	0.0009 (12)
O24	0.0448 (17)	0.0280 (14)	0.0382 (16)	−0.0118 (13)	−0.0002 (13)	0.0009 (12)
C73	0.057 (6)	0.050 (6)	0.053 (6)	−0.026 (5)	0.004 (5)	0.009 (5)
N13	0.084 (7)	0.083 (6)	0.079 (6)	−0.036 (6)	0.007 (6)	0.023 (6)
C74	0.089 (11)	0.098 (10)	0.072 (10)	−0.040 (10)	0.000 (9)	0.018 (9)
C75	0.097 (11)	0.116 (11)	0.092 (11)	−0.030 (10)	−0.008 (10)	0.032 (10)
O33	0.100 (8)	0.099 (7)	0.085 (7)	−0.042 (7)	0.017 (6)	0.018 (6)
O34	0.073 (7)	0.091 (7)	0.070 (7)	−0.056 (5)	−0.011 (5)	0.026 (5)
O25	0.053 (2)	0.0454 (18)	0.056 (2)	−0.0126 (16)	−0.0182 (17)	−0.0072 (16)
C49	0.058 (3)	0.045 (3)	0.064 (3)	−0.019 (2)	−0.019 (3)	−0.004 (2)
N5	0.053 (2)	0.057 (3)	0.067 (3)	−0.024 (2)	−0.023 (2)	0.014 (2)
C50	0.047 (3)	0.114 (6)	0.078 (4)	−0.006 (4)	−0.005 (3)	0.031 (4)
C51	0.092 (5)	0.077 (4)	0.077 (4)	−0.041 (4)	−0.040 (4)	0.017 (3)
O31	0.128 (6)	0.150 (6)	0.128 (6)	−0.099 (5)	0.031 (5)	−0.039 (5)
C67	0.108 (5)	0.105 (5)	0.071 (5)	−0.079 (4)	0.019 (4)	−0.014 (4)
N11	0.096 (5)	0.097 (4)	0.060 (4)	−0.073 (4)	0.006 (3)	0.004 (3)
C68	0.119 (7)	0.103 (6)	0.072 (5)	−0.082 (6)	0.018 (6)	−0.020 (5)
C69	0.084 (6)	0.085 (6)	0.109 (8)	−0.034 (5)	−0.010 (6)	0.021 (6)
O31B	0.132 (11)	0.143 (11)	0.108 (11)	−0.070 (10)	0.009 (10)	−0.009 (10)
C67B	0.108 (7)	0.114 (6)	0.076 (7)	−0.069 (6)	0.003 (6)	−0.015 (6)
N11B	0.111 (7)	0.108 (6)	0.075 (6)	−0.074 (6)	0.011 (6)	−0.010 (6)
C68B	0.105 (12)	0.109 (11)	0.084 (12)	−0.040 (11)	0.009 (12)	0.008 (11)
C69B	0.123 (13)	0.110 (11)	0.071 (11)	−0.061 (11)	0.011 (12)	0.000 (11)
Na1	0.0306 (8)	0.0275 (7)	0.0303 (8)	−0.0058 (6)	−0.0019 (6)	−0.0044 (6)
Mn1	0.0231 (3)	0.0161 (3)	0.0155 (3)	−0.0002 (2)	−0.0028 (2)	−0.0023 (2)
Mn2	0.0202 (3)	0.0180 (3)	0.0155 (3)	0.0006 (2)	−0.0024 (2)	−0.0015 (2)
Mn3	0.0203 (3)	0.0211 (3)	0.0140 (3)	0.0012 (2)	−0.0003 (2)	−0.0006 (2)
Mn4	0.0272 (3)	0.0174 (3)	0.0152 (3)	0.0012 (2)	−0.0008 (2)	0.0001 (2)
Y1	0.02044 (16)	0.01900 (16)	0.01589 (15)	−0.00179 (11)	−0.00074 (11)	−0.00104 (11)

### Geometric parameters (Å, º)

**Table:**

C1—O2	1.287 (4)	C74B—H74D	0.9800
C1—N1	1.313 (4)	C74B—H74E	0.9800
C1—C2	1.481 (5)	C74B—H74F	0.9800
C2—C7	1.404 (5)	C75B—H75D	0.9800
C2—C3	1.414 (5)	C75B—H75E	0.9800
C3—O3	1.329 (4)	C75B—H75F	0.9800
C3—C4	1.402 (5)	O29—C61	1.222 (9)
C4—C5	1.379 (5)	C61—N9	1.311 (8)
C4—H4	0.9500	C61—H61	0.9500
C5—C6	1.386 (6)	N9—C63	1.441 (9)
C5—H5	0.9500	N9—C62	1.460 (9)
C6—C7	1.385 (6)	C62—H62A	0.9800
C6—H6	0.9500	C62—H62B	0.9800
C7—H7	0.9500	C62—H62C	0.9800
C8—O5	1.288 (4)	C63—H63A	0.9800
C8—N2	1.320 (5)	C63—H63B	0.9800
C8—C9	1.475 (5)	C63—H63C	0.9800
C9—C14	1.408 (5)	O29B—C61B	1.245 (16)
C9—C10	1.417 (5)	C61B—N9B	1.310 (15)
C10—O6	1.332 (4)	C61B—H61B	0.9500
C10—C11	1.395 (5)	N9B—C63B	1.441 (15)
C11—C12	1.371 (6)	N9B—C62B	1.455 (15)
C11—H11	0.9500	C62B—H62D	0.9800
C12—C13	1.397 (6)	C62B—H62E	0.9800
C12—H12	0.9500	C62B—H62F	0.9800
C13—C14	1.381 (6)	C63B—H63D	0.9800
C13—H13	0.9500	C63B—H63E	0.9800
C14—H14	0.9500	C63B—H63F	0.9800
C15—O8	1.289 (4)	O30—C64	1.225 (7)
C15—N3	1.317 (5)	C64—N10	1.342 (7)
C15—C16	1.475 (5)	C64—H64	0.9500
C16—C21	1.409 (5)	N10—C65	1.450 (8)
C16—C17	1.414 (5)	N10—C66	1.453 (8)
C17—O9	1.329 (4)	C65—H65A	0.9800
C17—C18	1.408 (5)	C65—H65B	0.9800
C18—C19	1.376 (6)	C65—H65C	0.9800
C18—H18	0.9500	C66—H66A	0.9800
C19—C20	1.396 (6)	C66—H66B	0.9800
C19—H19	0.9500	C66—H66C	0.9800
C20—C21	1.383 (5)	O30B—C64B	1.247 (15)
C20—H20	0.9500	C64B—N10B	1.303 (14)
C21—H21	0.9500	C64B—H64B	0.9500
C22—O11	1.293 (4)	N10B—C66B	1.443 (14)
C22—N4	1.315 (4)	N10B—C65B	1.462 (15)
C22—C23	1.474 (5)	C65B—H65D	0.9800
C23—C28	1.406 (5)	C65B—H65E	0.9800
C23—C24	1.414 (5)	C65B—H65F	0.9800
C24—O12	1.332 (4)	C66B—H66D	0.9800
C24—C25	1.401 (5)	C66B—H66E	0.9800
C25—C26	1.369 (6)	C66B—H66F	0.9800
C25—H25	0.9500	O32—C70	1.258 (11)
C26—C27	1.393 (6)	C70—N12	1.319 (12)
C26—H26	0.9500	C70—H70	0.9500
C27—C28	1.378 (6)	N12—C71	1.467 (11)
C27—H27	0.9500	N12—C72	1.469 (11)
C28—H28	0.9500	C71—H71A	0.9800
C29—O14	1.251 (5)	C71—H71B	0.9800
C29—O13	1.261 (4)	C71—H71C	0.9800
C29—C30	1.537 (6)	C72—H72A	0.9800
C30—C32	1.521 (6)	C72—H72B	0.9800
C30—C31	1.522 (6)	C72—H72C	0.9800
C30—C33	1.538 (8)	O32B—C70B	1.250 (16)
C31—H31A	0.9800	C70B—N12B	1.323 (15)
C31—H31B	0.9800	C70B—H70B	0.9500
C31—H31C	0.9800	N12B—C72B	1.440 (16)
C32—H32A	0.9800	N12B—C71B	1.468 (16)
C32—H32B	0.9800	C71B—H71D	0.9800
C32—H32C	0.9800	C71B—H71E	0.9800
C33—H33A	0.9800	C71B—H71F	0.9800
C33—H33B	0.9800	C72B—H72D	0.9800
C33—H33C	0.9800	C72B—H72E	0.9800
C34—O16	1.251 (5)	C72B—H72F	0.9800
C34—O15	1.259 (5)	N1—O1	1.415 (4)
C34—C35	1.534 (6)	N1—Mn2	1.964 (3)
C35—C36	1.518 (7)	N2—O4	1.408 (4)
C35—C38	1.527 (6)	N2—Mn3	1.956 (3)
C35—C37	1.553 (7)	N3—O7	1.411 (4)
C36—H36A	0.9800	N3—Mn4	1.962 (3)
C36—H36B	0.9800	N4—O10	1.406 (4)
C36—H36C	0.9800	N4—Mn1	1.963 (3)
C37—H37A	0.9800	O1—Mn1	1.925 (2)
C37—H37B	0.9800	O1—Y1	2.439 (2)
C37—H37C	0.9800	O1—Na1	2.717 (3)
C38—H38A	0.9800	O2—Mn1	1.956 (2)
C38—H38B	0.9800	O3—Mn2	1.850 (3)
C38—H38C	0.9800	O4—Mn2	1.925 (2)
C39—O18	1.250 (5)	O4—Y1	2.419 (2)
C39—O17	1.274 (5)	O4—Na1	2.752 (3)
C39—C40	1.529 (6)	O5—Mn2	1.956 (2)
C40—C43	1.516 (7)	O6—Mn3	1.850 (3)
C40—C41	1.523 (6)	O7—Mn3	1.919 (2)
C40—C42	1.547 (8)	O7—Y1	2.429 (2)
C41—H41A	0.9800	O7—Na1	2.676 (3)
C41—H41B	0.9800	O8—Mn3	1.943 (2)
C41—H41C	0.9800	O9—Mn4	1.846 (3)
C42—H42A	0.9800	O10—Mn4	1.927 (2)
C42—H42B	0.9800	O10—Y1	2.427 (2)
C42—H42C	0.9800	O10—Na1	2.667 (3)
C43—H43A	0.9800	O11—Mn4	1.954 (2)
C43—H43B	0.9800	O12—Mn1	1.854 (3)
C43—H43C	0.9800	O13—Y1	2.261 (2)
C44—O20	1.248 (5)	O14—Mn1	2.140 (3)
C44—O19	1.271 (5)	O15—Y1	2.270 (2)
C44—C45	1.540 (6)	O16—Mn2	2.143 (3)
C45—C46	1.522 (6)	O17—Y1	2.281 (3)
C45—C48	1.526 (6)	O18—Mn3	2.132 (3)
C45—C47	1.542 (6)	O19—Y1	2.261 (2)
C46—H46A	0.9800	O20—Mn4	2.152 (3)
C46—H46B	0.9800	O21—Na1	2.460 (3)
C46—H46C	0.9800	O21—Mn1	2.466 (3)
C47—H47A	0.9800	O21—H21A	0.82 (2)
C47—H47B	0.9800	O21—H21B	0.83 (2)
C47—H47C	0.9800	O22—Mn2	2.423 (3)
C48—H48A	0.9800	O22—Na1	2.463 (3)
C48—H48B	0.9800	O22—H22A	0.84 (2)
C48—H48C	0.9800	O22—H22B	0.83 (2)
O26—C52	1.252 (6)	O23—Na1	2.449 (3)
C52—N6	1.312 (6)	O23—H23A	0.84 (2)
C52—H52	0.9500	O23—H23B	0.86 (2)
N6—C53	1.440 (8)	O24C—Na1	2.424 (3)
N6—C54	1.455 (7)	O24C—Mn4	2.469 (3)
C53—H53A	0.9800	O24C—H24A	0.863 (19)
C53—H53B	0.9800	O24C—H24B	0.859 (19)
C53—H53C	0.9800	O24—C73	1.251 (12)
C54—H54A	0.9800	O24—Na1	2.424 (3)
C54—H54B	0.9800	O24—Mn4	2.469 (3)
C54—H54C	0.9800	C73—N13	1.313 (14)
O27—C55	1.271 (9)	C73—H73	0.9500
C55—N7	1.298 (9)	N13—C75	1.447 (15)
C55—H55	0.9500	N13—C74	1.483 (15)
N7—C57	1.444 (10)	C74—H74A	0.9800
N7—C56	1.477 (10)	C74—H74B	0.9800
C56—H56A	0.9800	C74—H74C	0.9800
C56—H56B	0.9800	C75—H75A	0.9800
C56—H56C	0.9800	C75—H75B	0.9800
C57—H57A	0.9800	C75—H75C	0.9800
C57—H57B	0.9800	O33—H33E	0.8447
C57—H57C	0.9800	O33—H33F	0.8356
O27B—C55B	1.232 (6)	O34—H34A	0.8494
C55B—N7B	1.311 (6)	O34—H34B	0.8475
C55B—H55B	0.9500	O25—C49	1.232 (5)
N7B—C56B	1.453 (7)	C49—N5	1.311 (6)
N7B—C57B	1.460 (6)	C49—H49	0.9500
C56B—H56D	0.9800	N5—C50	1.452 (7)
C56B—H56E	0.9800	N5—C51	1.459 (6)
C56B—H56F	0.9800	C50—H50A	0.9800
C57B—H57D	0.9800	C50—H50B	0.9800
C57B—H57E	0.9800	C50—H50C	0.9800
C57B—H57F	0.9800	C51—H51A	0.9800
O28—C58	1.218 (8)	C51—H51B	0.9800
C58—N8	1.306 (9)	C51—H51C	0.9800
C58—H58	0.9500	O31—C67	1.284 (10)
N8—C60	1.439 (10)	C67—N11	1.304 (8)
N8—C59	1.476 (11)	C67—H67	0.9500
C59—H59A	0.9800	N11—C68	1.445 (9)
C59—H59B	0.9800	N11—C69	1.461 (10)
C59—H59C	0.9800	C68—H68A	0.9800
C60—H60A	0.9800	C68—H68B	0.9800
C60—H60B	0.9800	C68—H68C	0.9800
C60—H60C	0.9800	C69—H69A	0.9800
O28B—C58B	1.233 (15)	C69—H69B	0.9800
C58B—N8B	1.304 (14)	C69—H69C	0.9800
C58B—H58B	0.9500	O31B—C67B	1.232 (6)
N8B—C60B	1.444 (15)	C67B—N11B	1.311 (6)
N8B—C59B	1.455 (15)	C67B—H67B	0.9500
C59B—H59D	0.9800	N11B—C68B	1.452 (7)
C59B—H59E	0.9800	N11B—C69B	1.459 (6)
C59B—H59F	0.9800	C68B—H68D	0.9800
C60B—H60D	0.9800	C68B—H68E	0.9800
C60B—H60E	0.9800	C68B—H68F	0.9800
C60B—H60F	0.9800	C69B—H69D	0.9800
O24B—C73B	1.215 (15)	C69B—H69E	0.9800
C73B—N13B	1.290 (14)	C69B—H69F	0.9800
C73B—H73B	0.9500	Na1—Y1	3.5343 (15)
N13B—C75B	1.448 (15)	Na1—Mn4	3.6079 (16)
N13B—C74B	1.454 (15)	Na1—Mn3	3.6382 (15)
			
O2—C1—N1	121.6 (3)	O32—C70—N12	122.8 (13)
O2—C1—C2	119.4 (3)	O32—C70—H70	118.6
N1—C1—C2	119.0 (3)	N12—C70—H70	118.6
C7—C2—C3	119.6 (3)	C70—N12—C71	129.9 (11)
C7—C2—C1	117.7 (3)	C70—N12—C72	119.3 (10)
C3—C2—C1	122.8 (3)	C71—N12—C72	110.5 (11)
O3—C3—C4	117.6 (3)	N12—C71—H71A	109.5
O3—C3—C2	124.1 (3)	N12—C71—H71B	109.5
C4—C3—C2	118.2 (3)	H71A—C71—H71B	109.5
C5—C4—C3	121.1 (4)	N12—C71—H71C	109.5
C5—C4—H4	119.5	H71A—C71—H71C	109.5
C3—C4—H4	119.5	H71B—C71—H71C	109.5
C4—C5—C6	120.9 (3)	N12—C72—H72A	109.5
C4—C5—H5	119.5	N12—C72—H72B	109.5
C6—C5—H5	119.5	H72A—C72—H72B	109.5
C7—C6—C5	119.2 (4)	N12—C72—H72C	109.5
C7—C6—H6	120.4	H72A—C72—H72C	109.5
C5—C6—H6	120.4	H72B—C72—H72C	109.5
C6—C7—C2	121.0 (4)	O32B—C70B—N12B	120 (3)
C6—C7—H7	119.5	O32B—C70B—H70B	120.2
C2—C7—H7	119.5	N12B—C70B—H70B	120.2
O5—C8—N2	121.2 (3)	C70B—N12B—C72B	122 (3)
O5—C8—C9	119.8 (3)	C70B—N12B—C71B	117 (2)
N2—C8—C9	119.0 (3)	C72B—N12B—C71B	118 (2)
C14—C9—C10	118.9 (3)	N12B—C71B—H71D	109.5
C14—C9—C8	117.9 (3)	N12B—C71B—H71E	109.5
C10—C9—C8	123.2 (3)	H71D—C71B—H71E	109.5
O6—C10—C11	117.7 (3)	N12B—C71B—H71F	109.5
O6—C10—C9	123.7 (3)	H71D—C71B—H71F	109.5
C11—C10—C9	118.6 (3)	H71E—C71B—H71F	109.5
C12—C11—C10	121.5 (4)	N12B—C72B—H72D	109.5
C12—C11—H11	119.3	N12B—C72B—H72E	109.5
C10—C11—H11	119.3	H72D—C72B—H72E	109.5
C11—C12—C13	120.7 (4)	N12B—C72B—H72F	109.5
C11—C12—H12	119.7	H72D—C72B—H72F	109.5
C13—C12—H12	119.7	H72E—C72B—H72F	109.5
C14—C13—C12	118.9 (4)	C1—N1—O1	112.7 (3)
C14—C13—H13	120.6	C1—N1—Mn2	130.4 (2)
C12—C13—H13	120.6	O1—N1—Mn2	114.56 (19)
C13—C14—C9	121.4 (4)	C8—N2—O4	112.6 (3)
C13—C14—H14	119.3	C8—N2—Mn3	130.1 (2)
C9—C14—H14	119.3	O4—N2—Mn3	115.3 (2)
O8—C15—N3	121.0 (3)	C15—N3—O7	112.3 (3)
O8—C15—C16	119.3 (3)	C15—N3—Mn4	130.6 (2)
N3—C15—C16	119.7 (3)	O7—N3—Mn4	115.36 (19)
C21—C16—C17	119.6 (3)	C22—N4—O10	112.9 (3)
C21—C16—C15	117.9 (3)	C22—N4—Mn1	129.9 (2)
C17—C16—C15	122.5 (3)	O10—N4—Mn1	115.28 (19)
O9—C17—C18	117.5 (3)	N1—O1—Mn1	112.17 (18)
O9—C17—C16	124.5 (3)	N1—O1—Y1	121.55 (18)
C18—C17—C16	118.0 (3)	Mn1—O1—Y1	120.20 (11)
C19—C18—C17	121.3 (4)	N1—O1—Na1	106.95 (17)
C19—C18—H18	119.4	Mn1—O1—Na1	102.09 (10)
C17—C18—H18	119.4	Y1—O1—Na1	86.36 (8)
C18—C19—C20	121.0 (4)	C1—O2—Mn1	111.7 (2)
C18—C19—H19	119.5	C3—O3—Mn2	130.0 (2)
C20—C19—H19	119.5	N2—O4—Mn2	112.58 (18)
C21—C20—C19	118.9 (4)	N2—O4—Y1	121.43 (18)
C21—C20—H20	120.6	Mn2—O4—Y1	120.63 (11)
C19—C20—H20	120.6	N2—O4—Na1	104.91 (17)
C20—C21—C16	121.3 (4)	Mn2—O4—Na1	102.99 (10)
C20—C21—H21	119.4	Y1—O4—Na1	85.99 (8)
C16—C21—H21	119.4	C8—O5—Mn2	111.9 (2)
O11—C22—N4	121.0 (3)	C10—O6—Mn3	129.7 (2)
O11—C22—C23	119.4 (3)	N3—O7—Mn3	112.68 (18)
N4—C22—C23	119.6 (3)	N3—O7—Y1	121.91 (18)
C28—C23—C24	119.2 (3)	Mn3—O7—Y1	119.81 (11)
C28—C23—C22	117.9 (3)	N3—O7—Na1	103.38 (17)
C24—C23—C22	122.9 (3)	Mn3—O7—Na1	103.48 (10)
O12—C24—C25	117.7 (3)	Y1—O7—Na1	87.50 (8)
O12—C24—C23	124.1 (3)	C15—O8—Mn3	112.2 (2)
C25—C24—C23	118.2 (3)	C17—O9—Mn4	130.9 (2)
C26—C25—C24	121.3 (4)	N4—O10—Mn4	112.48 (18)
C26—C25—H25	119.3	N4—O10—Y1	121.36 (18)
C24—C25—H25	119.3	Mn4—O10—Y1	119.71 (11)
C25—C26—C27	121.0 (4)	N4—O10—Na1	106.01 (17)
C25—C26—H26	119.5	Mn4—O10—Na1	102.31 (10)
C27—C26—H26	119.5	Y1—O10—Na1	87.74 (8)
C28—C27—C26	118.8 (4)	C22—O11—Mn4	111.9 (2)
C28—C27—H27	120.6	C24—O12—Mn1	129.6 (2)
C26—C27—H27	120.6	C29—O13—Y1	139.9 (2)
C27—C28—C23	121.4 (4)	C29—O14—Mn1	123.2 (2)
C27—C28—H28	119.3	C34—O15—Y1	139.9 (2)
C23—C28—H28	119.3	C34—O16—Mn2	123.8 (2)
O14—C29—O13	124.8 (4)	C39—O17—Y1	140.9 (2)
O14—C29—C30	116.8 (3)	C39—O18—Mn3	124.7 (2)
O13—C29—C30	118.4 (3)	C44—O19—Y1	139.1 (2)
C32—C30—C31	110.1 (4)	C44—O20—Mn4	123.9 (2)
C32—C30—C29	109.5 (4)	Na1—O21—Mn1	95.42 (10)
C31—C30—C29	111.4 (4)	Na1—O21—H21A	116 (4)
C32—C30—C33	110.1 (4)	Mn1—O21—H21A	111 (4)
C31—C30—C33	109.4 (4)	Na1—O21—H21B	121 (4)
C29—C30—C33	106.2 (4)	Mn1—O21—H21B	114 (4)
C30—C31—H31A	109.5	H21A—O21—H21B	100 (5)
C30—C31—H31B	109.5	Mn2—O22—Na1	98.28 (11)
H31A—C31—H31B	109.5	Mn2—O22—H22A	118 (4)
C30—C31—H31C	109.5	Na1—O22—H22A	117 (4)
H31A—C31—H31C	109.5	Mn2—O22—H22B	122 (4)
H31B—C31—H31C	109.5	Na1—O22—H22B	116 (4)
C30—C32—H32A	109.5	H22A—O22—H22B	88 (5)
C30—C32—H32B	109.5	Na1—O23—H23A	133 (4)
H32A—C32—H32B	109.5	Na1—O23—H23B	115 (4)
C30—C32—H32C	109.5	H23A—O23—H23B	90 (5)
H32A—C32—H32C	109.5	Na1—O24C—Mn4	95.00 (10)
H32B—C32—H32C	109.5	Na1—O24C—H24A	115 (2)
C30—C33—H33A	109.5	Mn4—O24C—H24A	119 (4)
C30—C33—H33B	109.5	Na1—O24C—H24B	112.6 (19)
H33A—C33—H33B	109.5	Mn4—O24C—H24B	113 (4)
C30—C33—H33C	109.5	H24A—O24C—H24B	103 (3)
H33A—C33—H33C	109.5	C73—O24—Na1	157.0 (10)
H33B—C33—H33C	109.5	C73—O24—Mn4	108.0 (10)
O16—C34—O15	124.4 (4)	Na1—O24—Mn4	95.00 (10)
O16—C34—C35	116.8 (3)	O24—C73—N13	131.1 (17)
O15—C34—C35	118.8 (4)	O24—C73—H73	114.5
C36—C35—C38	110.3 (4)	N13—C73—H73	114.5
C36—C35—C34	109.2 (4)	C73—N13—C75	120.8 (19)
C38—C35—C34	111.7 (3)	C73—N13—C74	115.0 (18)
C36—C35—C37	110.5 (4)	C75—N13—C74	112.9 (19)
C38—C35—C37	109.0 (4)	N13—C74—H74A	109.5
C34—C35—C37	106.0 (3)	N13—C74—H74B	109.5
C35—C36—H36A	109.5	H74A—C74—H74B	109.5
C35—C36—H36B	109.5	N13—C74—H74C	109.5
H36A—C36—H36B	109.5	H74A—C74—H74C	109.5
C35—C36—H36C	109.5	H74B—C74—H74C	109.5
H36A—C36—H36C	109.5	N13—C75—H75A	109.5
H36B—C36—H36C	109.5	N13—C75—H75B	109.5
C35—C37—H37A	109.5	H75A—C75—H75B	109.5
C35—C37—H37B	109.5	N13—C75—H75C	109.5
H37A—C37—H37B	109.5	H75A—C75—H75C	109.5
C35—C37—H37C	109.5	H75B—C75—H75C	109.5
H37A—C37—H37C	109.5	H33E—O33—H33F	107.6
H37B—C37—H37C	109.5	H34A—O34—H34B	106.1
C35—C38—H38A	109.5	O25—C49—N5	125.1 (5)
C35—C38—H38B	109.5	O25—C49—H49	117.5
H38A—C38—H38B	109.5	N5—C49—H49	117.5
C35—C38—H38C	109.5	C49—N5—C50	119.1 (5)
H38A—C38—H38C	109.5	C49—N5—C51	121.8 (5)
H38B—C38—H38C	109.5	C50—N5—C51	119.0 (5)
O18—C39—O17	123.6 (4)	N5—C50—H50A	109.5
O18—C39—C40	117.6 (3)	N5—C50—H50B	109.5
O17—C39—C40	118.7 (3)	H50A—C50—H50B	109.5
C43—C40—C41	110.0 (4)	N5—C50—H50C	109.5
C43—C40—C39	109.3 (4)	H50A—C50—H50C	109.5
C41—C40—C39	112.9 (4)	H50B—C50—H50C	109.5
C43—C40—C42	109.9 (5)	N5—C51—H51A	109.5
C41—C40—C42	108.7 (4)	N5—C51—H51B	109.5
C39—C40—C42	105.8 (4)	H51A—C51—H51B	109.5
C40—C41—H41A	109.5	N5—C51—H51C	109.5
C40—C41—H41B	109.5	H51A—C51—H51C	109.5
H41A—C41—H41B	109.5	H51B—C51—H51C	109.5
C40—C41—H41C	109.5	O31—C67—N11	125.0 (9)
H41A—C41—H41C	109.5	O31—C67—H67	117.5
H41B—C41—H41C	109.5	N11—C67—H67	117.5
C40—C42—H42A	109.5	C67—N11—C68	125.4 (8)
C40—C42—H42B	109.5	C67—N11—C69	118.5 (8)
H42A—C42—H42B	109.5	C68—N11—C69	116.1 (7)
C40—C42—H42C	109.5	N11—C68—H68A	109.5
H42A—C42—H42C	109.5	N11—C68—H68B	109.5
H42B—C42—H42C	109.5	H68A—C68—H68B	109.5
C40—C43—H43A	109.5	N11—C68—H68C	109.5
C40—C43—H43B	109.5	H68A—C68—H68C	109.5
H43A—C43—H43B	109.5	H68B—C68—H68C	109.5
C40—C43—H43C	109.5	N11—C69—H69A	109.5
H43A—C43—H43C	109.5	N11—C69—H69B	109.5
H43B—C43—H43C	109.5	H69A—C69—H69B	109.5
O20—C44—O19	124.0 (4)	N11—C69—H69C	109.5
O20—C44—C45	117.9 (3)	H69A—C69—H69C	109.5
O19—C44—C45	118.2 (4)	H69B—C69—H69C	109.5
C46—C45—C48	110.2 (4)	O31B—C67B—N11B	125.1 (6)
C46—C45—C44	111.7 (3)	O31B—C67B—H67B	117.5
C48—C45—C44	109.6 (4)	N11B—C67B—H67B	117.5
C46—C45—C47	109.0 (4)	C67B—N11B—C68B	119.1 (5)
C48—C45—C47	110.1 (4)	C67B—N11B—C69B	121.9 (5)
C44—C45—C47	106.1 (3)	C68B—N11B—C69B	119.0 (5)
C45—C46—H46A	109.5	N11B—C68B—H68D	109.5
C45—C46—H46B	109.5	N11B—C68B—H68E	109.5
H46A—C46—H46B	109.5	H68D—C68B—H68E	109.5
C45—C46—H46C	109.5	N11B—C68B—H68F	109.5
H46A—C46—H46C	109.5	H68D—C68B—H68F	109.5
H46B—C46—H46C	109.5	H68E—C68B—H68F	109.5
C45—C47—H47A	109.5	N11B—C69B—H69D	109.5
C45—C47—H47B	109.5	N11B—C69B—H69E	109.5
H47A—C47—H47B	109.5	H69D—C69B—H69E	109.5
C45—C47—H47C	109.5	N11B—C69B—H69F	109.5
H47A—C47—H47C	109.5	H69D—C69B—H69F	109.5
H47B—C47—H47C	109.5	H69E—C69B—H69F	109.5
C45—C48—H48A	109.5	O24—Na1—O23	87.79 (11)
C45—C48—H48B	109.5	O24C—Na1—O23	87.79 (11)
H48A—C48—H48B	109.5	O24—Na1—O21	85.54 (11)
C45—C48—H48C	109.5	O24C—Na1—O21	85.54 (11)
H48A—C48—H48C	109.5	O23—Na1—O21	146.26 (12)
H48B—C48—H48C	109.5	O24—Na1—O22	149.78 (12)
O26—C52—N6	123.2 (5)	O24C—Na1—O22	149.78 (12)
O26—C52—H52	118.4	O23—Na1—O22	84.02 (11)
N6—C52—H52	118.4	O21—Na1—O22	85.33 (11)
C52—N6—C53	120.0 (5)	O24—Na1—O10	68.52 (9)
C52—N6—C54	121.3 (5)	O24C—Na1—O10	68.52 (9)
C53—N6—C54	118.6 (5)	O23—Na1—O10	124.35 (11)
N6—C53—H53A	109.5	O21—Na1—O10	83.38 (9)
N6—C53—H53B	109.5	O22—Na1—O10	138.49 (10)
H53A—C53—H53B	109.5	O24—Na1—O7	84.56 (10)
N6—C53—H53C	109.5	O24C—Na1—O7	84.56 (10)
H53A—C53—H53C	109.5	O23—Na1—O7	69.82 (10)
H53B—C53—H53C	109.5	O21—Na1—O7	141.90 (11)
N6—C54—H54A	109.5	O22—Na1—O7	119.17 (10)
N6—C54—H54B	109.5	O10—Na1—O7	58.78 (8)
H54A—C54—H54B	109.5	O24—Na1—O1	121.82 (10)
N6—C54—H54C	109.5	O24C—Na1—O1	121.82 (10)
H54A—C54—H54C	109.5	O23—Na1—O1	140.89 (11)
H54B—C54—H54C	109.5	O21—Na1—O1	67.70 (9)
O27—C55—N7	128.1 (9)	O22—Na1—O1	80.57 (9)
O27—C55—H55	116.0	O10—Na1—O1	58.19 (8)
N7—C55—H55	116.0	O7—Na1—O1	86.88 (8)
C55—N7—C57	123.4 (8)	O24—Na1—O4	142.11 (11)
C55—N7—C56	122.2 (9)	O24C—Na1—O4	142.11 (11)
C57—N7—C56	114.4 (8)	O23—Na1—O4	83.54 (10)
N7—C56—H56A	109.5	O21—Na1—O4	120.40 (10)
N7—C56—H56B	109.5	O22—Na1—O4	65.63 (9)
H56A—C56—H56B	109.5	O10—Na1—O4	86.37 (8)
N7—C56—H56C	109.5	O7—Na1—O4	57.82 (7)
H56A—C56—H56C	109.5	O1—Na1—O4	57.35 (7)
H56B—C56—H56C	109.5	O24—Na1—Y1	106.73 (9)
N7—C57—H57A	109.5	O24C—Na1—Y1	106.73 (9)
N7—C57—H57B	109.5	O23—Na1—Y1	107.52 (9)
H57A—C57—H57B	109.5	O21—Na1—Y1	106.08 (8)
N7—C57—H57C	109.5	O22—Na1—Y1	103.48 (8)
H57A—C57—H57C	109.5	O10—Na1—Y1	43.32 (5)
H57B—C57—H57C	109.5	O7—Na1—Y1	43.36 (5)
O27B—C55B—N7B	125.0 (6)	O1—Na1—Y1	43.53 (5)
O27B—C55B—H55B	117.5	O4—Na1—Y1	43.05 (5)
N7B—C55B—H55B	117.5	O24—Na1—Mn4	42.98 (8)
C55B—N7B—C56B	118.9 (5)	O24C—Na1—Mn4	42.98 (8)
C55B—N7B—C57B	121.7 (5)	O23—Na1—Mn4	99.01 (9)
C56B—N7B—C57B	118.8 (5)	O21—Na1—Mn4	98.38 (8)
N7B—C56B—H56D	109.5	O22—Na1—Mn4	167.24 (9)
N7B—C56B—H56E	109.5	O10—Na1—Mn4	31.45 (5)
H56D—C56B—H56E	109.5	O7—Na1—Mn4	51.71 (6)
N7B—C56B—H56F	109.5	O1—Na1—Mn4	89.51 (6)
H56D—C56B—H56F	109.5	O4—Na1—Mn4	102.24 (7)
H56E—C56B—H56F	109.5	Y1—Na1—Mn4	63.77 (3)
N7B—C57B—H57D	109.5	O24—Na1—Mn3	99.33 (8)
N7B—C57B—H57E	109.5	O24C—Na1—Mn3	99.33 (8)
H57D—C57B—H57E	109.5	O23—Na1—Mn3	44.12 (8)
N7B—C57B—H57F	109.5	O21—Na1—Mn3	169.26 (9)
H57D—C57B—H57F	109.5	O22—Na1—Mn3	94.84 (8)
H57E—C57B—H57F	109.5	O10—Na1—Mn3	89.48 (6)
O28—C58—N8	127.6 (7)	O7—Na1—Mn3	30.86 (5)
O28—C58—H58	116.2	O1—Na1—Mn3	101.70 (7)
N8—C58—H58	116.2	O4—Na1—Mn3	50.83 (5)
C58—N8—C60	126.4 (8)	Y1—Na1—Mn3	63.41 (3)
C58—N8—C59	117.5 (7)	Mn4—Na1—Mn3	79.24 (3)
C60—N8—C59	116.1 (7)	O12—Mn1—O1	172.51 (12)
N8—C59—H59A	109.5	O12—Mn1—O2	96.78 (11)
N8—C59—H59B	109.5	O1—Mn1—O2	81.85 (10)
H59A—C59—H59B	109.5	O12—Mn1—N4	90.93 (11)
N8—C59—H59C	109.5	O1—Mn1—N4	89.15 (11)
H59A—C59—H59C	109.5	O2—Mn1—N4	166.95 (11)
H59B—C59—H59C	109.5	O12—Mn1—O14	94.63 (11)
N8—C60—H60A	109.5	O1—Mn1—O14	92.76 (10)
N8—C60—H60B	109.5	O2—Mn1—O14	90.79 (11)
H60A—C60—H60B	109.5	N4—Mn1—O14	99.10 (11)
N8—C60—H60C	109.5	O12—Mn1—O21	91.16 (11)
H60A—C60—H60C	109.5	O1—Mn1—O21	81.40 (10)
H60B—C60—H60C	109.5	O2—Mn1—O21	85.59 (10)
O28B—C58B—N8B	126 (2)	N4—Mn1—O21	83.73 (11)
O28B—C58B—H58B	117.0	O14—Mn1—O21	173.51 (10)
N8B—C58B—H58B	117.0	O12—Mn1—Na1	126.89 (9)
C58B—N8B—C60B	120.2 (19)	O1—Mn1—Na1	46.81 (8)
C58B—N8B—C59B	124.0 (19)	O2—Mn1—Na1	101.65 (8)
C60B—N8B—C59B	115.9 (18)	N4—Mn1—Na1	65.33 (9)
N8B—C59B—H59D	109.5	O14—Mn1—Na1	133.92 (7)
N8B—C59B—H59E	109.5	O21—Mn1—Na1	42.23 (7)
H59D—C59B—H59E	109.5	O3—Mn2—O4	172.75 (12)
N8B—C59B—H59F	109.5	O3—Mn2—O5	96.84 (11)
H59D—C59B—H59F	109.5	O4—Mn2—O5	81.62 (10)
H59E—C59B—H59F	109.5	O3—Mn2—N1	90.58 (11)
N8B—C60B—H60D	109.5	O4—Mn2—N1	89.78 (11)
N8B—C60B—H60E	109.5	O5—Mn2—N1	167.69 (11)
H60D—C60B—H60E	109.5	O3—Mn2—O16	94.43 (11)
N8B—C60B—H60F	109.5	O4—Mn2—O16	92.66 (11)
H60D—C60B—H60F	109.5	O5—Mn2—O16	90.25 (11)
H60E—C60B—H60F	109.5	N1—Mn2—O16	98.98 (11)
O24B—C73B—N13B	136 (3)	O3—Mn2—O22	92.31 (11)
O24B—C73B—H73B	111.9	O4—Mn2—O22	80.55 (10)
N13B—C73B—H73B	111.9	O5—Mn2—O22	86.75 (10)
C73B—N13B—C75B	115.0 (18)	N1—Mn2—O22	83.14 (11)
C73B—N13B—C74B	126.8 (19)	O16—Mn2—O22	172.91 (10)
C75B—N13B—C74B	118.1 (18)	O3—Mn2—Na1	127.54 (9)
N13B—C74B—H74D	109.5	O4—Mn2—Na1	46.51 (8)
N13B—C74B—H74E	109.5	O5—Mn2—Na1	101.65 (8)
H74D—C74B—H74E	109.5	N1—Mn2—Na1	66.07 (8)
N13B—C74B—H74F	109.5	O16—Mn2—Na1	133.63 (8)
H74D—C74B—H74F	109.5	O22—Mn2—Na1	41.26 (7)
H74E—C74B—H74F	109.5	O6—Mn3—O7	170.54 (12)
N13B—C75B—H75D	109.5	O6—Mn3—O8	95.86 (11)
N13B—C75B—H75E	109.5	O7—Mn3—O8	81.69 (10)
H75D—C75B—H75E	109.5	O6—Mn3—N2	91.04 (11)
N13B—C75B—H75F	109.5	O7—Mn3—N2	89.58 (11)
H75D—C75B—H75F	109.5	O8—Mn3—N2	166.28 (12)
H75E—C75B—H75F	109.5	O6—Mn3—O18	95.75 (11)
O29—C61—N9	123.3 (7)	O7—Mn3—O18	93.46 (10)
O29—C61—H61	118.4	O8—Mn3—O18	91.75 (11)
N9—C61—H61	118.4	N2—Mn3—O18	99.36 (11)
C61—N9—C63	118.8 (7)	O6—Mn3—Na1	126.47 (9)
C61—N9—C62	122.6 (7)	O7—Mn3—Na1	45.66 (8)
C63—N9—C62	118.6 (8)	O8—Mn3—Na1	99.23 (8)
N9—C62—H62A	109.5	N2—Mn3—Na1	67.21 (9)
N9—C62—H62B	109.5	O18—Mn3—Na1	134.40 (7)
H62A—C62—H62B	109.5	O9—Mn4—O10	172.02 (12)
N9—C62—H62C	109.5	O9—Mn4—O11	97.27 (11)
H62A—C62—H62C	109.5	O10—Mn4—O11	81.63 (10)
H62B—C62—H62C	109.5	O9—Mn4—N3	90.42 (11)
N9—C63—H63A	109.5	O10—Mn4—N3	89.20 (11)
N9—C63—H63B	109.5	O11—Mn4—N3	166.41 (12)
H63A—C63—H63B	109.5	O9—Mn4—O20	94.64 (12)
N9—C63—H63C	109.5	O10—Mn4—O20	93.27 (10)
H63A—C63—H63C	109.5	O11—Mn4—O20	90.16 (11)
H63B—C63—H63C	109.5	N3—Mn4—O20	100.40 (12)
O29B—C61B—N9B	125 (3)	O9—Mn4—O24	91.44 (12)
O29B—C61B—H61B	117.4	O10—Mn4—O24	80.60 (10)
N9B—C61B—H61B	117.4	O11—Mn4—O24	85.58 (11)
C61B—N9B—C63B	120 (2)	N3—Mn4—O24	83.03 (11)
C61B—N9B—C62B	124 (2)	O20—Mn4—O24	172.98 (10)
C63B—N9B—C62B	114.4 (19)	O9—Mn4—O24C	91.44 (12)
N9B—C62B—H62D	109.5	O10—Mn4—O24C	80.60 (10)
N9B—C62B—H62E	109.5	O11—Mn4—O24C	85.58 (11)
H62D—C62B—H62E	109.5	N3—Mn4—O24C	83.03 (11)
N9B—C62B—H62F	109.5	O20—Mn4—O24C	172.98 (10)
H62D—C62B—H62F	109.5	O9—Mn4—Na1	126.87 (9)
H62E—C62B—H62F	109.5	O10—Mn4—Na1	46.24 (8)
N9B—C63B—H63D	109.5	O11—Mn4—Na1	101.43 (8)
N9B—C63B—H63E	109.5	N3—Mn4—Na1	65.06 (9)
H63D—C63B—H63E	109.5	O20—Mn4—Na1	134.11 (7)
N9B—C63B—H63F	109.5	O24—Mn4—Na1	42.02 (7)
H63D—C63B—H63F	109.5	O24C—Mn4—Na1	42.02 (7)
H63E—C63B—H63F	109.5	O19—Y1—O13	77.98 (9)
O30—C64—N10	126.2 (6)	O19—Y1—O15	123.27 (10)
O30—C64—H64	116.9	O13—Y1—O15	77.25 (9)
N10—C64—H64	116.9	O19—Y1—O17	76.42 (9)
C64—N10—C65	121.1 (6)	O13—Y1—O17	124.40 (10)
C64—N10—C66	122.1 (5)	O15—Y1—O17	77.15 (9)
C65—N10—C66	116.6 (6)	O19—Y1—O4	145.05 (9)
N10—C65—H65A	109.5	O13—Y1—O4	136.80 (8)
N10—C65—H65B	109.5	O15—Y1—O4	77.39 (9)
H65A—C65—H65B	109.5	O17—Y1—O4	82.48 (9)
N10—C65—H65C	109.5	O19—Y1—O10	78.54 (9)
H65A—C65—H65C	109.5	O13—Y1—O10	82.23 (9)
H65B—C65—H65C	109.5	O15—Y1—O10	145.10 (9)
N10—C66—H66A	109.5	O17—Y1—O10	137.52 (9)
N10—C66—H66B	109.5	O4—Y1—O10	99.89 (8)
H66A—C66—H66B	109.5	O19—Y1—O7	82.80 (9)
N10—C66—H66C	109.5	O13—Y1—O7	145.03 (8)
H66A—C66—H66C	109.5	O15—Y1—O7	137.39 (8)
H66B—C66—H66C	109.5	O17—Y1—O7	77.84 (9)
O30B—C64B—N10B	124 (2)	O4—Y1—O7	65.56 (8)
O30B—C64B—H64B	118.0	O10—Y1—O7	65.37 (8)
N10B—C64B—H64B	118.0	O19—Y1—O1	138.13 (8)
C64B—N10B—C66B	121.2 (18)	O13—Y1—O1	77.21 (9)
C64B—N10B—C65B	122.4 (19)	O15—Y1—O1	82.85 (9)
C66B—N10B—C65B	116.4 (19)	O17—Y1—O1	145.20 (9)
N10B—C65B—H65D	109.5	O4—Y1—O1	65.40 (8)
N10B—C65B—H65E	109.5	O10—Y1—O1	65.11 (8)
H65D—C65B—H65E	109.5	O7—Y1—O1	99.25 (8)
N10B—C65B—H65F	109.5	O19—Y1—Na1	117.64 (7)
H65D—C65B—H65F	109.5	O13—Y1—Na1	117.35 (7)
H65E—C65B—H65F	109.5	O15—Y1—Na1	119.08 (7)
N10B—C66B—H66D	109.5	O17—Y1—Na1	118.24 (7)
N10B—C66B—H66E	109.5	O4—Y1—Na1	50.95 (6)
H66D—C66B—H66E	109.5	O10—Y1—Na1	48.94 (6)
N10B—C66B—H66F	109.5	O7—Y1—Na1	49.15 (6)
H66D—C66B—H66F	109.5	O1—Y1—Na1	50.11 (6)
H66E—C66B—H66F	109.5		
			
O2—C1—C2—C7	−10.7 (5)	O32—C70—N12—C72	176.0 (13)
N1—C1—C2—C7	167.2 (3)	O32B—C70B—N12B—C72B	−134 (7)
O2—C1—C2—C3	170.0 (3)	O32B—C70B—N12B—C71B	25 (9)
N1—C1—C2—C3	−12.1 (5)	O2—C1—N1—O1	−1.9 (5)
C7—C2—C3—O3	175.8 (3)	C2—C1—N1—O1	−179.7 (3)
C1—C2—C3—O3	−4.9 (6)	O2—C1—N1—Mn2	−163.5 (2)
C7—C2—C3—C4	−2.3 (5)	C2—C1—N1—Mn2	18.6 (5)
C1—C2—C3—C4	177.0 (3)	O5—C8—N2—O4	−1.1 (5)
O3—C3—C4—C5	−177.0 (4)	C9—C8—N2—O4	−179.3 (3)
C2—C3—C4—C5	1.3 (6)	O5—C8—N2—Mn3	−164.1 (2)
C3—C4—C5—C6	0.6 (6)	C9—C8—N2—Mn3	17.7 (5)
C4—C5—C6—C7	−1.5 (6)	O8—C15—N3—O7	−2.7 (5)
C5—C6—C7—C2	0.4 (6)	C16—C15—N3—O7	178.6 (3)
C3—C2—C7—C6	1.5 (6)	O8—C15—N3—Mn4	−166.5 (3)
C1—C2—C7—C6	−177.8 (4)	C16—C15—N3—Mn4	14.8 (5)
O5—C8—C9—C14	−12.0 (5)	O11—C22—N4—O10	−1.3 (5)
N2—C8—C9—C14	166.3 (4)	C23—C22—N4—O10	−179.1 (3)
O5—C8—C9—C10	167.7 (3)	O11—C22—N4—Mn1	−164.5 (3)
N2—C8—C9—C10	−14.1 (5)	C23—C22—N4—Mn1	17.8 (5)
C14—C9—C10—O6	177.2 (4)	C1—N1—O1—Mn1	0.6 (3)
C8—C9—C10—O6	−2.4 (6)	Mn2—N1—O1—Mn1	165.36 (13)
C14—C9—C10—C11	−1.5 (6)	C1—N1—O1—Y1	153.2 (2)
C8—C9—C10—C11	178.9 (3)	Mn2—N1—O1—Y1	−42.1 (3)
O6—C10—C11—C12	−178.4 (4)	C1—N1—O1—Na1	−110.5 (3)
C9—C10—C11—C12	0.3 (6)	Mn2—N1—O1—Na1	54.2 (2)
C10—C11—C12—C13	0.9 (7)	N1—C1—O2—Mn1	2.1 (4)
C11—C12—C13—C14	−1.0 (7)	C2—C1—O2—Mn1	179.9 (2)
C12—C13—C14—C9	−0.2 (7)	C4—C3—O3—Mn2	−165.7 (3)
C10—C9—C14—C13	1.4 (6)	C2—C3—O3—Mn2	16.2 (5)
C8—C9—C14—C13	−178.9 (4)	C8—N2—O4—Mn2	−1.5 (3)
O8—C15—C16—C21	−7.5 (5)	Mn3—N2—O4—Mn2	164.14 (13)
N3—C15—C16—C21	171.1 (3)	C8—N2—O4—Y1	152.7 (2)
O8—C15—C16—C17	172.9 (3)	Mn3—N2—O4—Y1	−41.6 (3)
N3—C15—C16—C17	−8.4 (5)	C8—N2—O4—Na1	−112.8 (3)
C21—C16—C17—O9	177.8 (4)	Mn3—N2—O4—Na1	52.9 (2)
C15—C16—C17—O9	−2.7 (6)	N2—C8—O5—Mn2	3.2 (4)
C21—C16—C17—C18	−1.7 (6)	C9—C8—O5—Mn2	−178.6 (3)
C15—C16—C17—C18	177.8 (3)	C11—C10—O6—Mn3	−165.1 (3)
O9—C17—C18—C19	−179.2 (4)	C9—C10—O6—Mn3	16.3 (5)
C16—C17—C18—C19	0.3 (6)	C15—N3—O7—Mn3	0.7 (3)
C17—C18—C19—C20	0.9 (7)	Mn4—N3—O7—Mn3	167.18 (13)
C18—C19—C20—C21	−0.6 (7)	C15—N3—O7—Y1	154.2 (2)
C19—C20—C21—C16	−0.8 (7)	Mn4—N3—O7—Y1	−39.3 (3)
C17—C16—C21—C20	2.0 (6)	C15—N3—O7—Na1	−110.3 (3)
C15—C16—C21—C20	−177.5 (4)	Mn4—N3—O7—Na1	56.1 (2)
O11—C22—C23—C28	−11.8 (5)	N3—C15—O8—Mn3	3.3 (4)
N4—C22—C23—C28	166.0 (4)	C16—C15—O8—Mn3	−178.0 (3)
O11—C22—C23—C24	169.0 (3)	C18—C17—O9—Mn4	−172.5 (3)
N4—C22—C23—C24	−13.2 (6)	C16—C17—O9—Mn4	7.9 (6)
C28—C23—C24—O12	177.2 (4)	C22—N4—O10—Mn4	−0.6 (3)
C22—C23—C24—O12	−3.6 (6)	Mn1—N4—O10—Mn4	165.20 (13)
C28—C23—C24—C25	−1.1 (6)	C22—N4—O10—Y1	151.1 (2)
C22—C23—C24—C25	178.1 (4)	Mn1—N4—O10—Y1	−43.1 (3)
O12—C24—C25—C26	−177.5 (4)	C22—N4—O10—Na1	−111.6 (3)
C23—C24—C25—C26	0.8 (6)	Mn1—N4—O10—Na1	54.2 (2)
C24—C25—C26—C27	0.1 (7)	N4—C22—O11—Mn4	2.5 (4)
C25—C26—C27—C28	−0.8 (7)	C23—C22—O11—Mn4	−179.8 (3)
C26—C27—C28—C23	0.5 (7)	C25—C24—O12—Mn1	−165.3 (3)
C24—C23—C28—C27	0.4 (6)	C23—C24—O12—Mn1	16.5 (5)
C22—C23—C28—C27	−178.8 (4)	O14—C29—O13—Y1	−60.1 (6)
O14—C29—C30—C32	−47.8 (6)	C30—C29—O13—Y1	117.9 (4)
O13—C29—C30—C32	134.0 (4)	O13—C29—O14—Mn1	16.7 (5)
O14—C29—C30—C31	−169.9 (4)	C30—C29—O14—Mn1	−161.3 (3)
O13—C29—C30—C31	11.9 (6)	O16—C34—O15—Y1	−59.2 (6)
O14—C29—C30—C33	71.0 (5)	C35—C34—O15—Y1	118.9 (4)
O13—C29—C30—C33	−107.1 (4)	O15—C34—O16—Mn2	18.4 (5)
O16—C34—C35—C36	−45.5 (5)	C35—C34—O16—Mn2	−159.8 (3)
O15—C34—C35—C36	136.2 (4)	O18—C39—O17—Y1	−54.7 (6)
O16—C34—C35—C38	−167.8 (4)	C40—C39—O17—Y1	126.3 (4)
O15—C34—C35—C38	13.9 (5)	O17—C39—O18—Mn3	12.7 (5)
O16—C34—C35—C37	73.6 (5)	C40—C39—O18—Mn3	−168.3 (3)
O15—C34—C35—C37	−104.7 (4)	O20—C44—O19—Y1	−60.2 (6)
O18—C39—C40—C43	−47.7 (6)	C45—C44—O19—Y1	119.3 (4)
O17—C39—C40—C43	131.4 (4)	O19—C44—O20—Mn4	17.2 (5)
O18—C39—C40—C41	−170.5 (4)	C45—C44—O20—Mn4	−162.3 (3)
O17—C39—C40—C41	8.5 (6)	Na1—O24—C73—N13	−63 (4)
O18—C39—C40—C42	70.7 (5)	Mn4—O24—C73—N13	116 (3)
O17—C39—C40—C42	−110.3 (4)	O24—C73—N13—C75	155 (3)
O20—C44—C45—C46	−169.2 (4)	O24—C73—N13—C74	14 (4)
O19—C44—C45—C46	11.2 (5)	O25—C49—N5—C50	2.1 (8)
O20—C44—C45—C48	−46.8 (5)	O25—C49—N5—C51	178.0 (5)
O19—C44—C45—C48	133.6 (4)	O31—C67—N11—C68	11.3 (16)
O20—C44—C45—C47	72.0 (4)	O31—C67—N11—C69	−169.6 (10)
O19—C44—C45—C47	−107.5 (4)	O31B—C67B—N11B—C68B	−0.1 (4)
O26—C52—N6—C53	2.1 (7)	O31B—C67B—N11B—C69B	−180.0 (4)
O26—C52—N6—C54	179.3 (5)	C24—O12—Mn1—O2	179.9 (3)
O27—C55—N7—C57	176.1 (11)	C24—O12—Mn1—N4	−10.7 (3)
O27—C55—N7—C56	−6.7 (18)	C24—O12—Mn1—O14	88.5 (3)
O27B—C55B—N7B—C56B	−10 (4)	C24—O12—Mn1—O21	−94.4 (3)
O27B—C55B—N7B—C57B	179 (3)	C24—O12—Mn1—Na1	−70.0 (3)
O28—C58—N8—C60	175.8 (8)	C3—O3—Mn2—O5	−179.4 (3)
O28—C58—N8—C59	−1.4 (12)	C3—O3—Mn2—N1	−9.2 (3)
O28B—C58B—N8B—C60B	180.0 (4)	C3—O3—Mn2—O16	89.8 (3)
O28B—C58B—N8B—C59B	0.0 (4)	C3—O3—Mn2—O22	−92.4 (3)
O24B—C73B—N13B—C75B	179.9 (4)	C3—O3—Mn2—Na1	−69.0 (3)
O24B—C73B—N13B—C74B	0.1 (5)	C10—O6—Mn3—O8	−179.4 (3)
O29—C61—N9—C63	−176.1 (8)	C10—O6—Mn3—N2	−11.3 (3)
O29—C61—N9—C62	2.7 (13)	C10—O6—Mn3—O18	88.2 (3)
O29B—C61B—N9B—C63B	−131 (5)	C10—O6—Mn3—Na1	−73.4 (3)
O29B—C61B—N9B—C62B	64 (7)	C17—O9—Mn4—O11	−171.4 (3)
O30—C64—N10—C65	5.1 (11)	C17—O9—Mn4—N3	−2.7 (3)
O30—C64—N10—C66	179.9 (7)	C17—O9—Mn4—O20	97.8 (3)
O30B—C64B—N10B—C66B	171 (4)	C17—O9—Mn4—O24	−85.7 (3)
O30B—C64B—N10B—C65B	−13 (6)	C17—O9—Mn4—O24C	−85.7 (3)
O32—C70—N12—C71	3 (2)	C17—O9—Mn4—Na1	−61.2 (4)

### Hydrogen-bond geometry (Å, º)

**Table:**

*D*—H···*A*	*D*—H	H···*A*	*D*···*A*	*D*—H···*A*
C18—H18···O20^i^	0.95	2.60	3.359 (5)	137
C25—H25···O14^ii^	0.95	2.59	3.374 (5)	141
C49—H49···O29	0.95	2.58	3.180 (8)	121
C51—H51*B*···O29^iii^	0.98	2.56	3.376 (9)	141
C53—H53*B*···O31^iv^	0.98	2.48	3.377 (9)	152
C55—H55···O8	0.95	2.36	3.098 (8)	135
C56—H56*A*···O32^iv^	0.98	2.56	3.499 (17)	162
C59—H59*B*···O29	0.98	2.56	3.262 (11)	129
C61—H61···O12	0.95	2.52	3.457 (8)	169
C63*B*—H63*F*···O32*B*^iii^	0.98	2.53	3.34 (6)	140
C64*B*—H64*B*···O3	0.95	2.50	3.40 (3)	157
C71*B*—H71*D*···O21	0.98	2.60	3.41 (5)	141
C72*B*—H72*E*···O34^iv^	0.98	2.36	3.31 (7)	163
C74—H74*B*···O27	0.98	2.27	2.87 (3)	119
C75—H75*C*···O31	0.98	2.15	2.99 (3)	143
O21—H21*A*···O25	0.82 (2)	2.00 (3)	2.767 (4)	155 (5)
O21—H21*B*···O28	0.83 (2)	2.05 (3)	2.792 (5)	148 (5)
O21—H21*B*···O28*B*	0.83 (2)	1.87 (3)	2.70 (2)	172 (5)
O22—H22*A*···O25	0.84 (2)	1.96 (3)	2.727 (4)	151 (5)
O22—H22*B*···O26	0.83 (2)	1.93 (3)	2.688 (4)	151 (5)
O23—H23*A*···O27	0.84 (2)	2.06 (3)	2.871 (7)	164 (5)
O23—H23*A*···O24*B*	0.84 (2)	2.06 (5)	2.696 (19)	132 (5)
O23—H23*B*···O26	0.86 (2)	1.98 (3)	2.789 (5)	155 (5)
O24*C*—H24*A*···O33	0.86 (2)	1.91 (4)	2.78 (3)	179 (5)

Symmetry codes: (i) −*x*+2, −*y*+1, −*z*+1; (ii) −*x*+2, −*y*+1, −*z*; (iii) −*x*+1, −*y*+1, −*z*; (iv) −*x*+1, −*y*+1, −*z*+1.
